# Balanced Impurity and Interfacial Effects in La-Loaded ZrS_2_/Co_*x*_Ni_*y*_O(OH)_*z*_ Create Key Characteristics for Photocatalytic Water Splitting in DFT Simulation

**DOI:** 10.1021/acsphyschemau.5c00128

**Published:** 2026-03-15

**Authors:** Joran Celis, Aku Lempelto, Wei Cao

**Affiliations:** Nano and Molecular Systems Research Unit, Faculty of Science, University of Oulu, Oulu FIN-90014, Finland

**Keywords:** photocatalyst by design, water splitting, density functional theory, charge-transfer scheme, band bending, heterojunction

## Abstract

Water splitting photocatalysts can be tracked in first-principles simulation. This involves probing versatile series of changes to atomistic models until the calculated properties morph to perfection. In this article, a design process as such unfolds. First, a prescriptive guideline is derived to obtain semiconductive derivatives of the CoO_2_ monolayer. This inspires the drawing of a set of bilayers consisting of CoO_2_ on ZrS_2_, with H, La, and Ni impurities. Precisely tuned redox potentials and a supportive built-in electric field manifest in DFT simulations when these impurities are balanced in appropriate ratios. It is shown how charge redistributions and the formed interlayer and intralayer dipoles underpin this outcome. The LaZr_16_S_32_/Co_14_Ni_14_O_45_(OH)_11_ bilayer is identified as a promising water splitting photocatalyst. The system is remarkable because of its polarized semihybridized single-gap electronic structure. Initial prospects on mechanistic properties are also provided, suggesting that 3Ni-coordinated oxygen promotes water splitting through enhanced vacancy formation. The work provides conceptual enrichment for any researcher in the field of photocatalysis.

## Graphical abstract

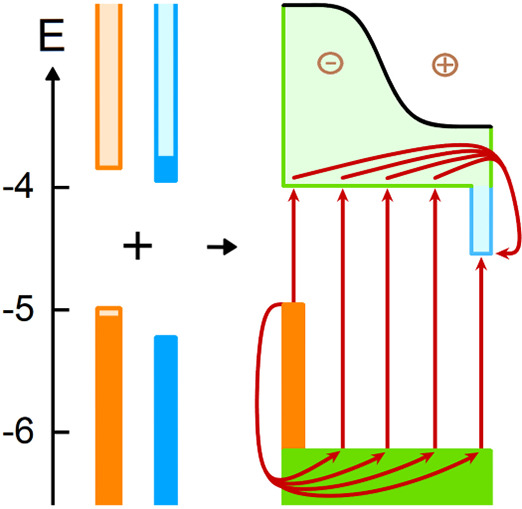

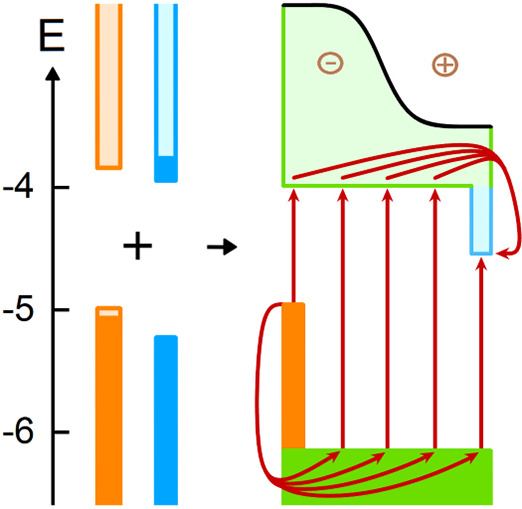

## Introduction

Clean and sustainable energy generation could make the world a little bit brighter. A detailed exploration of technical means for this purpose is appropriate and is still very much ongoing in both industry and academia.^1,2^ Included in this effort is the mastery of photocatalytic water splitting,^3,4^ which essentially involves light absorption by a catalyst material and H_2_O splicing to produce oxygen and hydrogen gases. The concept is particularly captivating as a means of sunlight energy harvesting. However, suitable low-cost and highly efficient photocatalyst materials remain lacking despite decades of research on the technology.^5^

In the present understanding of the topic, photocatalysis is considered a sequence of separate subprocesses, and its overall efficiency, a multiplication of their separate efficiencies.^4,6^ Slight variations in terms of compartmentalization into subprocesses have been articulated.^4,6–9^ On this occasion, photon absorption, charge carrier transfer, and chemical reaction are considered the three elementary steps of photocatalysis. Their concerted optimization within a highly multifunctional material structure appears as an optimal strategy for photocatalyst innovation.^4,6^

This viewpoint translates into concrete photocatalyst material demands. Assumed is that a pH of 14 evokes the alkaline reaction mechanism under standard conditions (eqs 1–3).^10^ Excited electrons of a photocatalyst are then thermodynamically required an energy above −3.61 eV with respect to vacuum to advance the hydrogen evolution reaction (HER) (eq 1).^11–13^ On the other hand, holes require an energy below −4.84 eV with respect to vacuum to further the oxygen evolution reaction (OER) (eq 2).

$$2H_{2}O+2e^{-}\to 2OH^{-}+H_{2}$$ 1

$$2OH^{-}\to 1/2O_{2}+H_{2}O+2e^{-}$$ 2

$$H_{2}O\to H_{2}+1/2O_{2}$$ 3

In addition, it is preferred that the photocatalyst is a multicomponent semiconducting system where OER and HER reaction sites are positioned in separate compartments for two important reasons.^14^ The first is the possible inclusion of a component with a smaller bandgap than the minimum gap of a functional single-component system. Lower energy photons can then be absorbed and in turn may contribute to light harvesting. The fraction of sunlight energy to be potentially tapped into is then set to increase.^5,9^ Furthermore, multiple photocatalyst compartments imply the formation of interfaces. Those may benefit photocatalysis by establishing built-in electric fields (BIEF) that directionally funnel charge carriers across the interface.^4,5,15,16^ In this way, charge separation proceeds, whereas electron–hole recombination is curbed. The probability of photogenerated charge carriers being consumed in chemical processes increases. Next, the photocatalyst material is required to be sufficiently stable under operating conditions.^8,17^ It also likely benefits from a high surface area and a high density of active sites.^18^ Finally, there are kinetic requirements for a photocatalyst, which essentially boil down to the activation energy of the rate-limiting step over the minimum energy reaction pathway.^7^

The above theoretical demands may be invaluable for guiding experimental photocatalysis research forward. However, they are merely of a general nature and do not directly reveal which nanostructural features or specific stoichiometry are best pursued. A bridge from general to atomically precise guidelines may be accomplished by density functional theory (DFT) calculations.^3,17,19^ Indeed, very many quantities can be calculated for atomistic models, which, when outlined to the photocatalyst demands, provide a prospect on the photocatalytic performance of real materials.^20,21^ Since structural variations can be introduced in these models simply by hand, their impacts are readily verified. In this way, systems can be perfected toward photocatalytic water splitting on a trial-and-error basis, representing photocatalysts by design.

In this paper, a water splitting photocatalyst is proposed on the basis of a design trajectory. The derived system includes a laterally stacked CoO_2_ monolayer on a ZrS_2_ monolayer together with H, Ni, and La impurities. The separate components of the system have been previously dealt with in the scientific literature to some extent. In fact, the CoO_2_ monolayer, its hydrated phases of CoO­(OH) and Co­(OH)_2_, and nickel-included CoNi hydroxide were reported as active OER catalysts by experiment.^22–25^ ZrS_2_ is known to be synthetically achievable in monolayer form^26,27^ and has been considered as a promising HER catalyst in many other bilayer systems by DFT calculations.^28–38^ Despite the above, ZrS_2_ and CoO_2_ were never reported together in a heterostructure before to the best of our knowledge. Furthermore, all elements used share the important aspect of cheap pricing, with the exception of medium-priced lanthanum.^39^ Finally, it can be noted that a variety of lanthanum-based water splitting catalysts and cocatalysts have been investigated.^40^

## Results and Discussion

### Semiconductive CoO_2_ Derivatives

As a point of departure, the impacts of H, Ni, and La impurities on the electronic structure of the CoO_2_ monolayer are first discussed. The pristine CoO_2_ monolayer was previously revealed to be a half-metal,^41–44^ meaning that the Fermi level is positioned inside the bandgap in the spin-up channel and within the valence band in the spin-down channel.^41–44^ Strategies to modify the CoO_2_ monolayer to create semiconductive properties have sparked interest, in line with the viewpoint that water splitting photocatalysts are preferably semiconductors. Hence, several derivatives of a Co_24_O_48_ slab model were probed, where H atoms or La atoms were adsorbed, or Ni atoms were doped (Figure 1).

**Figure 1:**
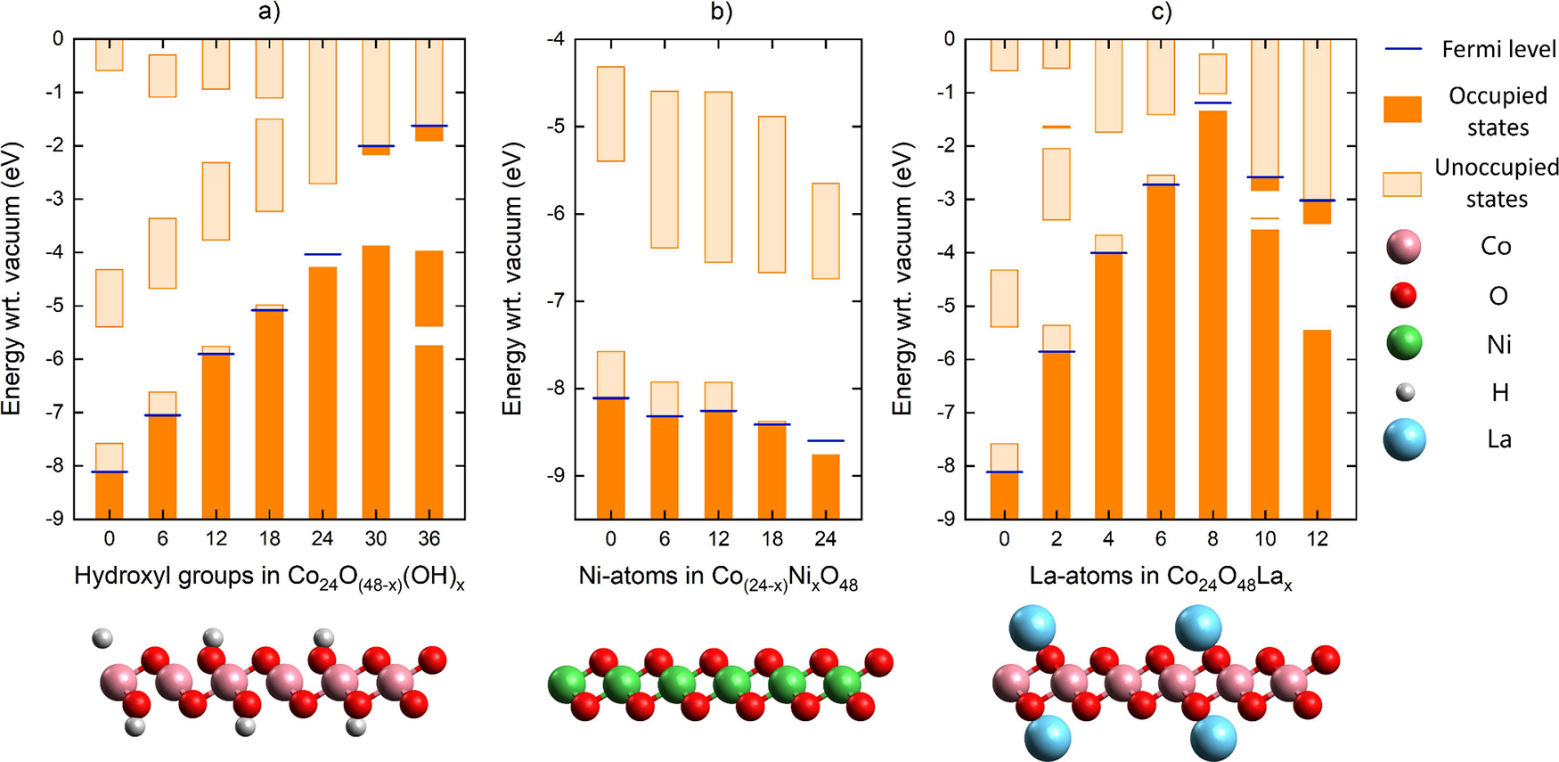
Schematically represented nonspin-polarized band structures of CoO_2_ monolayer and (a) hydrogen adsorbed, (b) nickel doped, and (c) lanthanum adsorbed.

Nonspin-polarized calculations show that the partially filled valence band of the pristine CoO_2_ monolayer gradually fills with increasing impurity concentrations (Figure 1a–c). Then, a metal-to-semiconductor transition occurs as NiO_2_, CoOOH, and La_0.33_CoO_2_ stoichiometries form. This is in line with previous literature where NiO_2_ and CoOOH monolayers were reported as semiconductors.^43–47^ When impurities are added beyond the CoOOH and La_0.33_CoO_2_ stoichiometries, the lower part of the conduction band becomes also occupied. This highlights the electron-donating character of each of the impurities.

$$N_{\mathrm{Co}}=N_{H}+3N_{\mathrm{La}}$$ 4

Furthermore, the stoichiometries at which the metal-to-semiconductor transition completes can be noted to follow eq 4 in the above results, where *N*_X_ is the number of atoms of element X in the simulation cell. This finding may be rationalized straightforwardly: Nickel possesses an additional electron compared to cobalt, hydrogen atoms add an electron to the system, and lanthanum firmly prefers a +3 oxidation state in oxidic compounds. Nevertheless, to validate the generality of eq 4, additional CoO_2_ derivatives with mixed impurities were modeled, specifically, Co_24_O_48_H_6_La_6_, Co_18_O_48_Ni_6_La_6_, Co_12_O_48_Ni_12_, and Co_14_O_48_Ni_10_H_8_La_2_. For all just-mentioned systems, it turns out that eq 4 holds. They were indeed calculated to be semiconductors (Figure 2).

**Figure 2:**
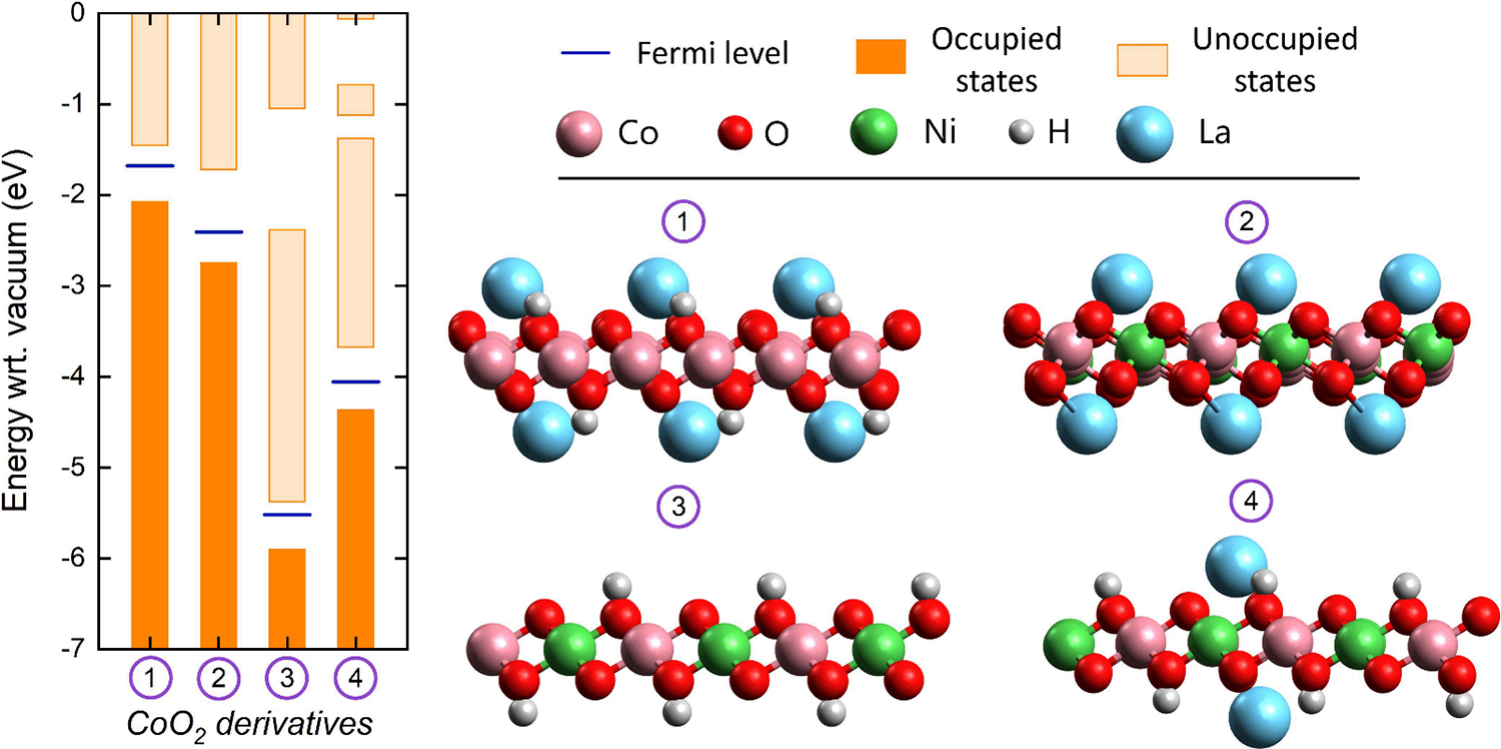
Schematically represented nonspin-polarized band structures of the CoO_2_ monolayer with mixed impurities.

However, the metal-to-semiconductor transition in Figure 1 could not be reproduced when including spin polarization. Instead, nearly all systems were calculated to be semiconductors. This is a consequence of the simplified effective Hubbard *U* correction that generally imposes band splitting in the considered half-metals, which is likely an unphysical feature. Metal-to-semiconductor transitions have been observed in Na_*x*_CoO_2_ and Li_*y*_CoO_2_ crystals in experiments, in line with the trend of the nonspin-polarized calculations.^42,48–51^ A more detailed discussion on this aspect is provided in Supplement S1. Moving forward, eq 4 is applied as a way of guiding modifications to CoO_2_ in order to create semiconductive systems.

The results from the spin-polarized calculations still yield insight into magnetism. The cell magnetic moments were found to be minor or completely zero for CoO_2_ derivatives adhering to eq 4. Other systems had a cell magnetic moment in the order of N_Co_–N_H_–3N_La_ μ_B_. This suggests that semiconductivity and diamagnetism coincide.

In addition, notice how the band edge positions among semiconducting CoO_2_ derivatives show strong variations with the impurities. The inclusion of lanthanum leads to the steepest increase in the band edges with respect to vacuum, followed by hydrogen. Nickel doping causes a slight decrease in the band edges with respect to vacuum. It suggests that the redox potentials of CoO_2_ derivatives could be deliberately tailored via the choice of impurities to precisely adhere to the demands of photocatalytic water splitting.

### Photocatalytic Bilayer Designs

The fulfilment of eq 4 to yield semiconductive properties was the first of a few design principles used in pursuit of a promising photocatalyst system. Second, another monolayer material was investigated as a counterpart to a CoO_2_ derivative. This was in the understanding that at least one separate material component and a BIEF across the interface are essential drivers of photocatalysis. Here, a ZrS_2_ monolayer was chosen as the counterpart. Initially, the impacts of lanthanum adsorption on the electronic structure of a Zr_24_S_48_ model were checked. The electron-donating character of lanthanum, previously encountered in the CoO_2_ derivatives, reemerged. Since ZrS_2_ is a known semiconductor in its pristine form, the outer electrons of the adsorbed lanthanum atom are placed into the conduction band of the ZrS_2_ material (Figure 3a).

**Figure 3:**
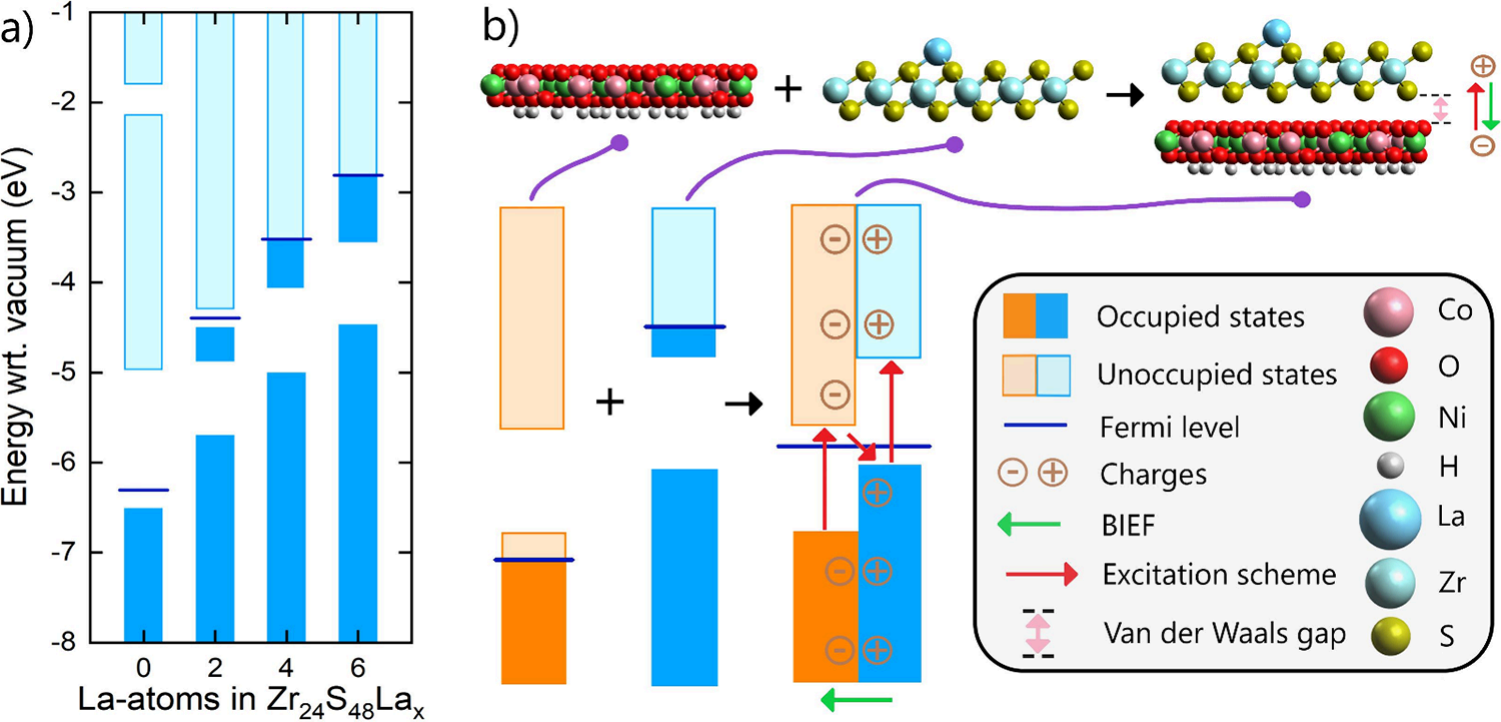
(a) Schematic band structures of ZrS_2_ with adsorbed lanthanum on both sides of the monolayer and (b) hypothesized BIEF formation across a bilayer La@ZrS_2_/Co_*x*_Ni_*y*_O­(OH)_*z*_ system.

With this result in mind, it can be expected that the interaction between La@ZrS_2_ and half-metallic Co_*x*_Ni_*y*_O­(OH)_*z*_ features an electron density redistribution from the La@ZrS_2_ conduction band to the Co_*x*_Ni_*y*_O­(OH)_*z*_ valence band (Figure 3b). Accordingly, charges in the ground state of the composite system would create a BIEF orientated from La@ZrS_2_ pointing toward Co_*x*_Ni_*y*_O­(OH)_*z*_. Then, under illumination, the accumulation of holes in Co_*x*_Ni_*y*_O­(OH)_*z*_ and excited electrons in La@ZrS_2_ would become favored, stimulating OER and HER, respectively.

As the last design principle, H was adsorbed only at the bottom of a lower Co_*x*_Ni_*y*_O_2_ layer and La was adsorbed only at the top of an upper ZrS_2_ layer. The purpose was to base the attractive force between the La@ZrS_2_ and Co_*x*_Ni_*y*_O­(OH)_*z*_ layers mainly on weak van der Waals interactions.^32,52,53^ Then, the interlayer distance of the bilayer would become accordingly large (Figure 3b), which spatially separates the system into two distinct compartments. The bilayer may hence be approached as a bicomponent system, rather than a single monolayer material.

Under these design principles, plausible systems still differ from each other by impurity concentrations. The systems defined by the H and La concentrations shown in Figure 4a,b and Table S1 were chosen from this material space to be modeled by DFT. Notice that the system variations were defined only by the concentrations of H and La because a corresponding amount of Ni is implied by eq 4. Also, notice that the data point at zero H and La concentration refers to a pristine ZrS_2_/NiO_2_ bilayer. The trends of many photocatalysis-related features of the bilayers are presented in the following sections. In addition, a first assessment of dynamic stability is discussed on the basis of phonon dispersion calculations in Supplement S3 (Figure S4).

**Figure 4:**
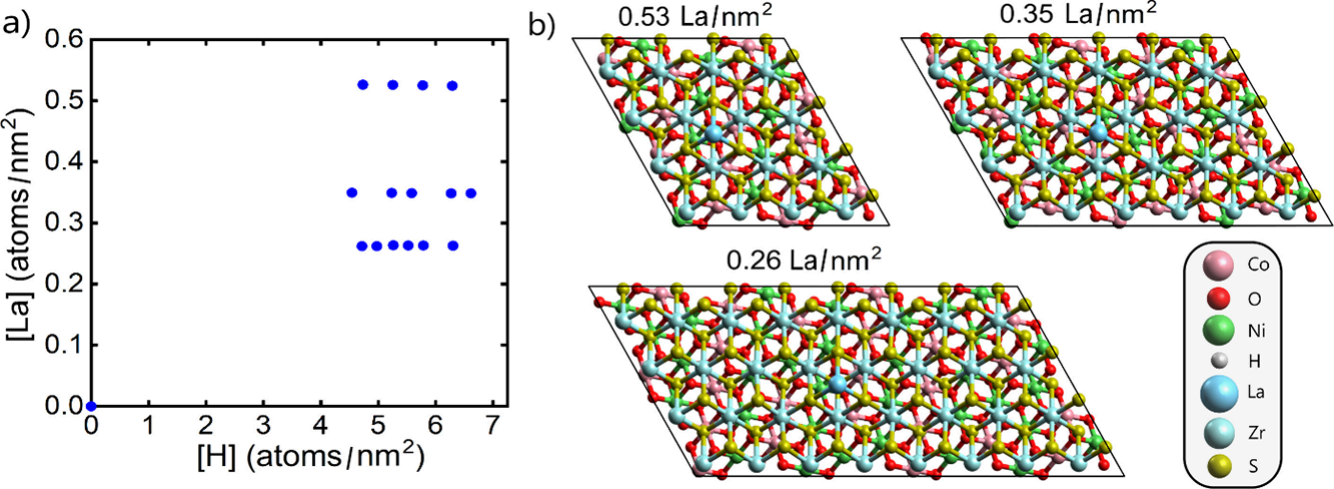
(a) Surface concentrations of H and La impurities in modeled La@ZrS_2_/Co_*x*_Ni_*y*_O­(OH)_*z*_ systems and (b) top view of modeled La@Zr_16_S_32_/Co_14_Ni_14_O_45_(OH)_11_, La@Zr_24_S_48_/Co_19_Ni_23_O_68_(OH)_16_, and La@Zr_32_S_64_/Co_23_Ni_33_O_92_(OH)_20_.

### Dipole-Related Properties

Bader analysis was applied to partition the electron densities of the optimized bilayers into atomic and layer-partitioned charges. An interlayer charge redistribution of 0.29 *e* nm^–2^ is calculated for the ZrS_2_/NiO_2_ reference, redistributing electron density from the ZrS_2_ layer to the NiO_2_ layer upon bilayer formation. The adsorption of La comes alongside the formation of a near-constant positive atomic charge on La, between 1.79 and 1.82 *e*. Across the layers, electron density is redistributed from La@ZrS_2_ to Co_*x*_Ni_*y*_O­(OH)_*z*_ with increasing La concentration (Figure 5a). It is seen that 0.77 *e* nm^–2^ is redistributed at 0.26 La atoms/nm^2^, up to 1.27 *e* nm^–2^ at 0.53 La atoms/nm^2^. This is a characteristic of BIEFs that support photocatalytic water splitting, as hypothesized in Figure 3b.

**Figure 5:**
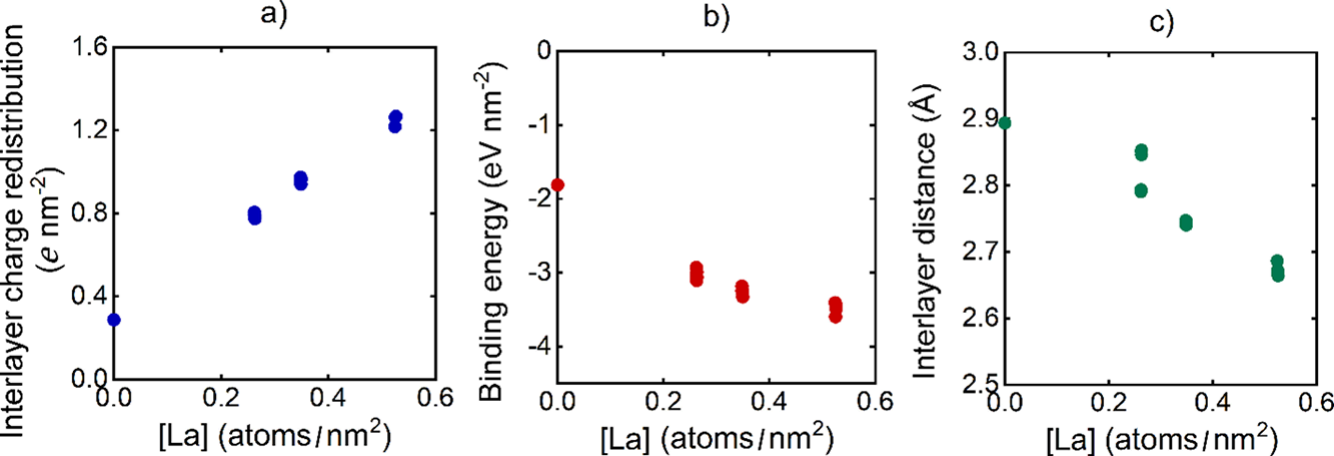
(a) Interlayer charge redistribution, (b) binding energy, and (c) interlayer distance for the modeled La@ZrS_2_/Co_*x*_Ni_*y*_O­(OH)_*z*_ systems.

The binding energies (*E*_b_) between La@ZrS_2_ and Co_*x*_Ni_*y*_O­(OH)_*z*_ correlate with the interlayer charge redistribution (Figure 5b). *E*_b_ takes a magnitude of −1.81 eV nm^–2^ for the ZrS_2_/NiO_2_ reference, which can be considered ordinary within the class of van der Waals heterostructures.^54^ Then, it is increased with the La concentration to between −2.93 and −3.60 eV nm^–2^. It suggests that interlayer charge redistribution leads to an attractive Coulombic force between the layers, which strengthens interlayer bonding. This is also apparent from the geometries of the optimized bilayers, more specifically from interlayer distances (*d*_0_) (Figure 5c). Stronger interactions are accompanied by shorter *d*_0_ values. *d*_0_ is retrieved at 2.89 Å for the ZrS_2_/NiO_2_ reference and between 2.85 and 2.66 Å for the La@ZrS_2_/Co_*x*_Ni_*y*_O­(OH)_*z*_ bilayers. All of the above quantities appear to be unaffected by H concentration.

The interlayer charge redistribution can also be expressed in terms of an accompanied interlayer dipole moment. However, the latter does not solely dictate the total dipole moment of the bilayer. Intralayer dipole moments appear across the Co_*x*_Ni_*y*_O­(OH)_*z*_ monolayers, separately, and range between 1.43 and 2.00 *e* Å nm^–2^, depending linearly on the H concentration (Figure 6b). In separate La@ZrS_2_ monolayers, intralayer dipole moments are present in the opposite direction with magnitudes between −0.38 and −0.75 *e* Å nm^–2^, depending linearly on La concentration (Figure 6c). The interlayer dipole moment is then obtained by subtracting the total dipole moment of the bilayer by the sum of the dipole moments in the monolayers separately. In this way, the interlayer dipoles are found between −1.01 and −1.54 *e* Å nm^–2^ (Figure 6d). They point in the same direction compared to the intralayer dipole on La@ZrS_2_ and are linearly correlated with the interlayer charge redistribution.

**Figure 6:**
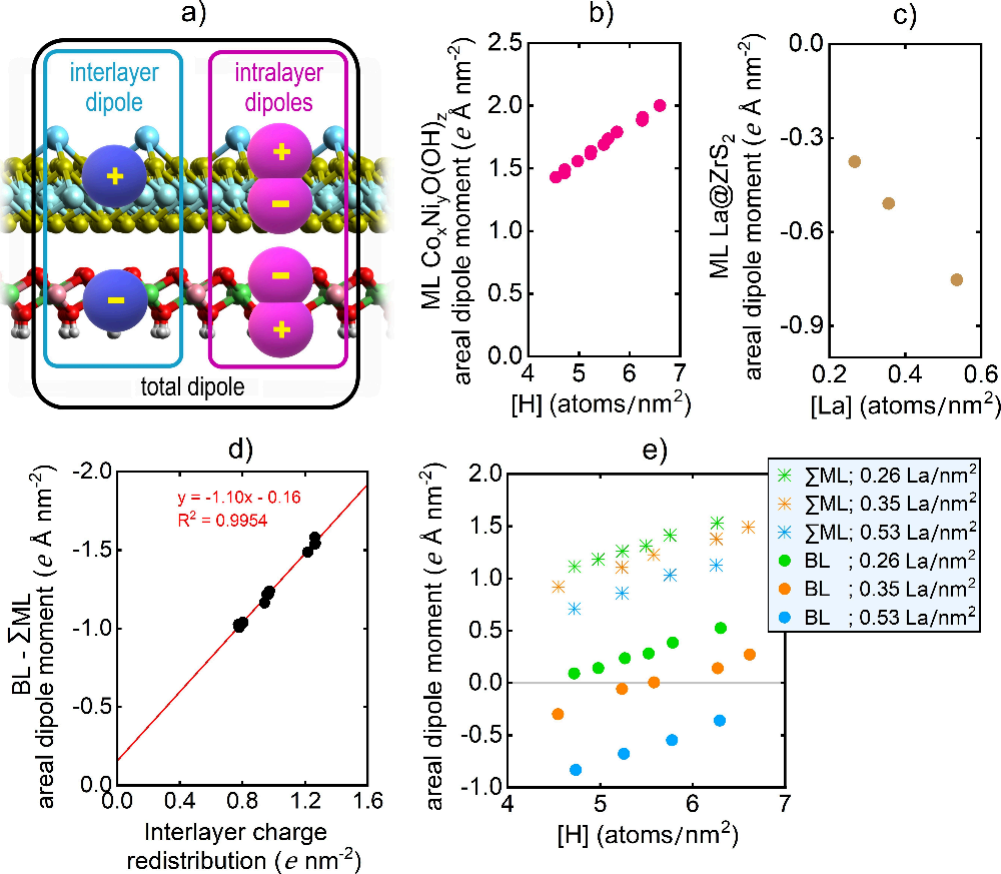
(a) Schematic representation of the dipoles across the bilayers. (b, c) Calculated intralayer dipole moments. (d) Calculated interlayer dipole moments. (e) Total dipole moments on La@ZrS_2_/Co_*x*_Ni_*y*_O­(OH)_*z*_ systems.

Lastly, the total dipole moments across the bilayers are calculated between −0.83 and (+)­0.52 *e* Å nm^–2^. Thus, the orientation of the total dipole moment flips with changing H and La concentrations as they govern the interlayer and intralayer dipole moments (Figure 6e). This affects photocatalysis. The total dipole moment possesses a perfect linear relationship to the vacuum energy change across the bilayer (Δ*E*_vac_ = *E*_vac,upper_ – *E*_vac,lower_) as clarified in Supplement S4 (Figure S5). It is precisely these upper and lower vacuum levels that serve as reference points when redox potentials are assigned to the electronic states. The lower vacuum is used as the reference to assign the oxidation potential of Co_*x*_Ni_*y*_O­(OH)_*z*_ for OER. The upper vacuum is used as the reference to assign the reduction potential of La@ZrS_2_ for HER. In line with the total dipole moments, we find Δ*E*_vac_ varying between −1.50 and 0.97 eV among the tested bilayers.

### Redox Potentials

To examine the redox potentials of the bilayers against impurity concentrations, we first address that a substantial part of electronic states is delocalized over both layers simultaneously, in spite of the presence of the van der Waals gap. This finding has been encountered in other works before^55–57^ and may be related to the ionic character of the monolayers that make up the bilayers.^58^ Still, validity remains when dealing with La@ZrS_2_/Co_*x*_Ni_*y*_O­(OH)_*z*_ as composites made of separate compartments, since the electronic states at the band edges consistently localize at only one of the two layers.

Our approach for dealing with hybridizations in the assessment of redox potentials is justified using the schematic model outcome in Figure 7. It roughly illustrates the features in the calculated distributions of electronic states of La@ZrS_2_/Co_*x*_Ni_*y*_O­(OH)_*z*_ in terms of energy and layer-projected occupation. Electronic states are classified as Co_*x*_Ni_*y*_O­(OH)_*z*_ or La@ZrS_2_ states when the layer-projected occupation exceeds 87.5%. A valence band maximum (VBM) and a conduction band minimum (CBM) are consistently assigned to Co_*x*_Ni_*y*_O­(OH)_*z*_ as such. However, this required omitting spin polarization, which otherwise introduced a small number of obscuring midgap states in some cases. In the case of the La@ZrS_2_ layer, a valence band could not be assigned due to overall hybridization with Co_*x*_Ni_*y*_O­(OH)_*z*_ below the Fermi level (*E*_F_). Above *E*_F_, the electronic states localized onto La@ZrS_2_ into either one or two conduction bands, defining CBM_1_ and CBM_2_.

**Figure 7:**
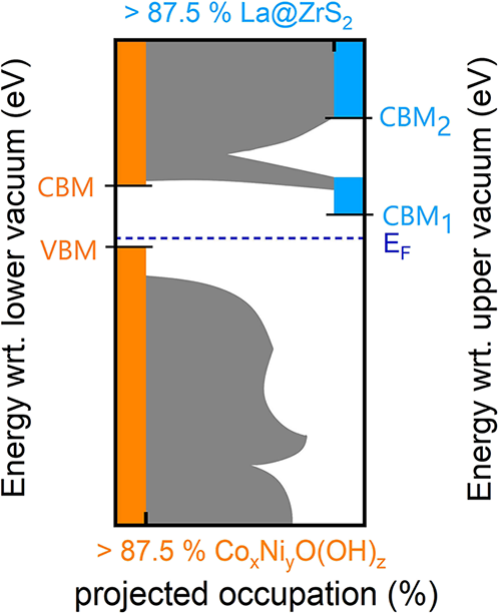
Schematic model outcome, roughly depicting the energies of the electronic states against their layer-projected occupation for La@ZrS_2_/Co_*x*_Ni_*y*_O­(OH)_*z*_ bilayers.

For the bilayers tested, the VBM of Co_*x*_Ni_*y*_O­(OH)_*z*_ steadily increases from −5.63 to −4.46 eV with increasing H concentration (Figure 8a). This range also contains the ideal oxidation potential for OER in an alkaline environment of −4.84 eV. H concentrations between 5.23 and 5.78 atoms/nm^2^ may be considered excellent for photocatalytic water splitting. It is also seen that the band edge positions of Co_*x*_Ni_*y*_O­(OH)_*z*_ are rather unaffected by bilayer formation. The VBM positions of Co_*x*_Ni_*y*_O­(OH)_*z*_ in the bilayers are on average 0.11 eV higher compared to the separate monolayers. The CBM positions of Co_*x*_Ni_*y*_O­(OH)_*z*_ in the bilayers are on average 0.09 eV lower compared to the separate monolayers. Thus, the band gap size is reduced on average by 0.20 eV due to bilayer formation.

**Figure 8:**
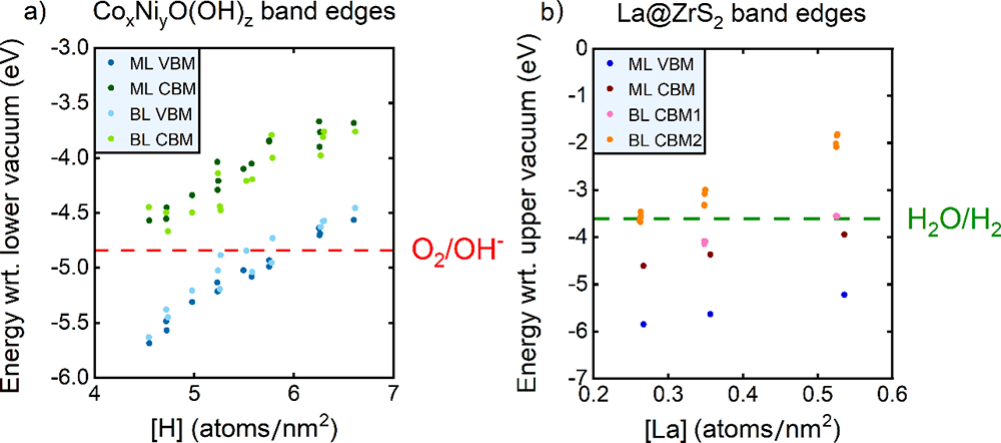
(a) Band edges of Co_*x*_Ni_*y*_O­(OH)_*z*_ for various H concentrations referenced to the lower vacuum level from nonspin-polarized calculations. (b) Band edges of La@ZrS_2_ for various La concentrations referenced to the upper vacuum level from spin-polarized calculations.

CBM_1_ and CBM_2_ of La@ZrS_2_ in the bilayers increase with La concentration (Figure 8b). Consequently, either the CBM_1_ or the CBM_2_ value becomes suitable for HER at each of the three tested La concentrations. At 0.26 and 0.35 La atoms/nm^2^, CBM_2_ is found at −3.59 and −3.15 eV, respectively. At 0.53 La atoms/nm^2^, CBM_1_ is found at −3.56 eV. Recall that the reduction potential for HER in an alkaline environment is ideally slightly above −3.61 eV. Thus, the highest reduction potential on the separate La@ZrS_2_ monolayers, at −3.94 eV, is deemed insufficient. The change in the reduction potential of La@ZrS_2_ due to bilayer formation is especially substantial when we consider that the CBM of the separate monolayer corresponds to CBM_2_ of the bilayer. Then, the band edge positions increase by 0.93, to 2.13 eV, due to bilayer formation. This finding can be rationalized considering that the adsorption of H and La and the interlayer dipole formation have separate effects on the band edge positions. Thereby, the shifts of the band edges are in the same direction for La@ZrS_2_ and in opposite directions for Co_*x*_Ni_*y*_O­(OH)_*z*_, which explains the contrasting outcomes.

From the viewpoint of the band edges given above, the three most favorable systems for photocatalytic water splitting are identified, one for each of the tested La concentrations. Some of the calculated properties of these three bilayers are summarized in Table 1. A top view of these systems is included as Figure 4b. Furthermore, the materials’ capability for light absorption was estimated using the calculated dielectric function in Supplement S5 (Figure S6). The simulated spectra show a broad range of adsorption for all systems with a peak around 2.7 eV, corresponding to blue light. In what follows, the bilayer’s redox abilities are analyzed in greater depth through the fully comprehensive view of electronic states in terms of energy and layer-projected occupations, shown in Figure 9.

**Table 1:** Summary of Key Properties of the Three Most Favorable La@ZrS_2_/Co_*x*_Ni_*y*_O­(OH)_*z*_ Bilayers for Photocatalytic Water Splitting, from Spin-Polarized Calculations

stoichiometry	VBM (eV)	CBM (eV)	CBM type	interlayer charge redistribution (*e* nm^–2^)	total dipole moment (*e* Å nm^–2^)	Δ*E*_vac_ (eV)
LaZr_32_S_64_/Co_23_Ni_33_O_92_(OH)_20_	–4.92	–3.56	2	0.78	+0.24	+0.44
LaZr_24_S_48_/Co_19_Ni_23_O_68_(OH)_16_	–5.04	–3.08	2	0.96	+0.00	+0.01
LaZr_16_S_32_/Co_14_Ni_14_O_45_(OH)_11_	–4.95	–3.55	1	1.26	–0.55	–0.99

**Figure 9:**
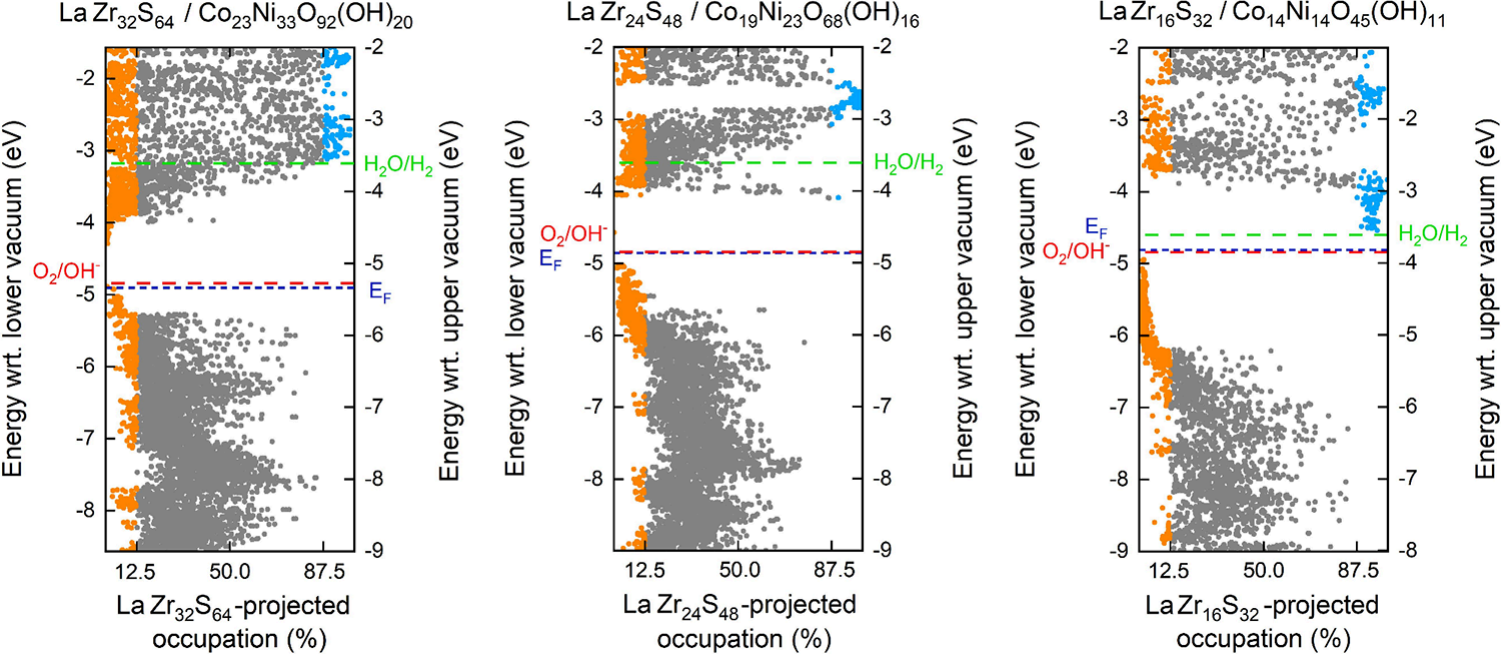
Spin-polarized electronic structures from the perspective of layer-projected occupations of three La@ZrS_2_/Co_*x*_Ni_*y*_O­(OH)_*z*_ bilayers with favorable redox potentials for photocatalytic water splitting.

Although the redox potentials are precisely tuned to the demands of alkaline photocatalytic water splitting for all three bilayers, the gaps between the ideal O_2_/OH^–^ and H_2_O/H_2_ redox potentials, depicted in Figure 9, differ greatly. This corresponds to the total dipole field, which either demands or supplies energy to an electron traveling from the lower vacuum to the upper vacuum. From this point of view, the LaZr_16_S_32_/Co_14_Ni_14_O_45_(OH)_11_ system is the most favorable, supplying −0.99 eV to a traversing electron. Across the LaZr_32_S_64_/Co_23_Ni_33_O_92_(OH)_20_ bilayer, the energy of a traversing electron is adversely affected by 0.44 eV.

Another remarkable difference among bilayers in Figure 9 is the localization of the electronic states at the bottom of the conduction band. The situation is intermediary on LaZr_24_S_48_/Co_19_Ni_23_O_68_(OH)_16_, where virtually any layer-projected occupation is obtained. On the other hand, the lower interlayer dipole in LaZr_32_S_64_/Co_23_Ni_33_O_92_(OH)_20_ is consistent with the complete localization of the CBM on Co_23_Ni_33_O_92_(OH)_20_ and the stronger interlayer dipole in LaZr_16_S_32_/Co_14_Ni_14_O_45_(OH)_11_ is consistent with the complete localization of the CBM on LaZr_16_S_32_. This outcome can be understood as a consequence of the interlayer charge redistribution impacting the electronic potential in a way that bends the shape of the optimized electronic states toward occupation on La@ZrS_2_ over Co_*x*_Ni_*y*_O­(OH)_*z*_. This idea is further elaborated in Supplement S6 via the *z*-plane averaged potential distributions (Figures S7 and S8). Nevertheless, the outcome can be suspected to benefit photocatalytic water splitting on LaZr_16_S_32_/Co_14_Ni_14_O_45_(OH)_11_. Only for this system, each excitation across the bandgap can be expected to generate a localized hole on Co_*x*_Ni_*y*_O­(OH)_*z*_ and a localized excited electron on La@ZrS_2_. This is under the assumption that rapid relaxations guide the photoinduced charges to the global VBM and the global CBM.^5^ From this point of view, LaZr_16_S_32_/Co_14_Ni_14_O_45_(OH)_11_ may be noted to offer truly excellent prospects for photocatalytic water splitting.

### Mechanistic Properties of Water Splitting on La@ZrS_2_/Co_*x*_Ni_*y*_O­(OH)_*z*_

We take an additional look at some of the key mechanistic properties of water splitting by using the three favorable systems (Table 1) that were identified in the previous section. The formation of oxygen vacancies on the surfaces of catalyst systems is often thought to be a source of improved catalytic activity.^24,25^ Therefore, their stability on the surface can be indicative of a good catalyst. We also used the binding and dissociation of water—the first steps in HER—as simple preliminary descriptors of catalytic ability.

We first examine the thermodynamics of vacancy formation on the hydroxide component. The vacancy formation energies Δ*E*_Ov_ calculated according to eq 5 (see Table 2) are in the order of +1.2 to +2.1 eV and therefore quite reasonable and in line with those of similar materials reported in the past.^25^ The creation of the oxygen defect is affected by the closest cations as it is easiest at sites surrounded by nickel cations and becomes progressively more endothermic for oxygen sites with one to three Co cations. For example, on the La@Zr_24_S_48_/Co_19_Ni_23_O_68_(OH)_16_ system, the Δ*E*_Ov_ goes from +1.24 eV for a 3-Ni-coordinated oxygen to +2.93 eV for a 3-Co-coordinated oxygen. The clear difference in vacancy formation energies seen in Table 2 between the second system and the others is due to the presence of these 3-Ni-coordinated sites. However, the energies are very similar (±0.2 eV) at sites with equivalent local surroundings regardless of the system.

**Table 2:** Oxygen Vacancy Formation Energy and Strongest Adsorption Energies of Water Molecules at Different Sites^a^

		Δ*E*_ads_ (H_2_O) [eV]		
system	Δ*E*_Ov_ [eV]	Co_*x*_Ni_*y*_O(OH)_*z*_	La@ZrS_2_	Co_*x*_Ni_*y*_O(OH)_*z*_ + O_v_
La@Zr_16_S_32_/Co_14_Ni_14_O_45_(OH)_11_	+2.11	–1.16	–1.65	–1.41
La@Zr_24_S_48_/Co_19_Ni_23_O_68_(OH)_16_	+1.24	–1.14	–1.69	–0.95
La@Zr_32_S_64_/Co_23_Ni_33_O_92_(OH)_20_	+2.05	–1.10	–1.45	–1.23

a The oxygen vacancy formation energy is given for the most stable vacancy on the hydroxide surface. The last columns represent adsorption on the neat hydroxide (Co_*x*_Ni_*y*_O­(OH)_*z*_), hydroxide with oxygen vacancy (Co_*x*_Ni_*y*_O­(OH)_*z*_ + Ov), and the La center on the ZrS_2_ surface (La@ZrS_2_). The adsorption energies are determined based on spin-polarized calculations.

The vacancy formation energies calculated for the isolated monolayer can also be compared with those of the bilayer system, revealing a small stabilization of oxygen vacancies upon bilayer formation with La@ZrS_2_. The vacancy formation energy on the Co_19_Ni_23_O_68_(OH)_16_ monolayer is +1.49 eV, which is approximately 0.25 eV more endothermic than in the equivalent combined system. To some extent, this can be rationalized by the redistribution of charge from La@ZrS_2_ to the hydroxide that is observed in the bilayer structures.

The calculated adsorption energies of water molecules that have been structurally relaxed at different sites of the three bilayered systems can be seen in Table 2. Note that the following discussion of adsorbate binding can be interpreted as applying to all three when not otherwise stated. Let us begin by examining the Co_*x*_Ni_*y*_O­(OH)_*z*_ monolayer as the site for H_2_O adsorption and dissociation. In the most favorable adsorption geometry (see Figure S9b for an example), the oxygen of the H_2_O molecule forms a hydrogen bond (∼1.7 Å) with a surface lattice hydroxyl while its hydrogens show a preference for pointing toward adjacent unhydrogenated surface oxygen sites. The adsorption energy of molecular water on the side of the hydroxyl ranges between −0.8 and −1.2 eV depending on the specific site. Sites that are surrounded by more Ni cations than Co are favored by a small margin. Although the water molecule does not bind directly to the oxygen vacancies on the hydroxide monolayer, it seems to strengthen adsorption in the vicinity by approximately 0.1–0.3 eV. The exception to this is the La@Zr_24_S_48_/Co_19_Ni_23_O_68_(OH)_16_ system where adsorption near the vacancy is slightly less strong. The lack of an increase in reactivity further speaks to the stability of the vacancy at the nickel-surrounded site.

The dissociative adsorption of water on the surface of any of the neat Co_*x*_Ni_*y*_O­(OH)_*z*_ systems in Table 1 is not favorable. Based on our simulations, it is unlikely that water stays in its dissociated form on the hydrogen-covered Co_*x*_Ni_*y*_O­(OH)_*z*_ surfaces, and the loose OH species instead grabs a nearby hydrogen and spontaneously reverts to an H_2_O molecule during geometry relaxation. Dehydrogenation of the hydroxide surface, which could occur, e.g., under basic conditions, creates sites where the dissociation products become (meta)­stable, as surface sites that are sufficiently separated become available. However, the energy required to split an adsorbed molecule remains around 0.7–1.0 eV until roughly three-quarters of the surface hydrogens have been removed, at which point the splitting is slightly exothermic at −0.3 eV or thermoneutral. The precise dissociation energies (Table S3) do not change monotonically with the number of surface hydroxyls, presumably because they are subtly affected by complicated underlying factors.

Reaction free energies Δ*G*_r_ for the water splitting reaction *H_2_O → *OH + H^+^(aq) + e^–^ after dehydrogenation of the La@Zr_24_S_48_/Co_19_Ni_23_O_68_(OH)_16_ system can be found in Table S2. The reaction free energies range from 1.96 to 0.68 eV (pH = 14, *U* = 0), corresponding to reduction potentials of ca. −0.7 to −2 V needed to shift the equilibrium toward the products. While reaction barriers were not explicitly calculated, the Brønsted-Evans–Polanyi principle, which has been applied successfully to catalytic surface reactions,^59,60^ suggests that the less endothermic reaction energies would also correspond to manageable reaction barriers.

Furthermore, an oxygen vacancy on the surface enables a stable dissociated form of water. The molecule is split into an OH group, which fills the vacancy and a surface-bound hydrogen that is positioned on an adjacent O top site (see, e.g., Figure S9e). In the La@Zr_16_S_32_/Co_14_Ni_14_O_45_(OH)_11_ and La@Zr_32_S_64_/Co_23_Ni_33_O_92_(OH)_20_ systems, the dissociation at the vacancy is clearly exothermic at −0.78 and −0.28 eV, respectively. In the case of the La@Zr_24_S_48_/Co_19_Ni_23_O_68_(OH)_16_ system, where vacancy formation energy was significantly lower, this dissociation is practically thermoneutral. Nevertheless, the fact that the vacancies create sites where the dissociation products are stable reinforces the idea that they are important for catalytic activity. The calculated reaction free energy of water splitting at the vacancy is 0.18 eV, which is clearly lower than even on the dehydrogenated surfaces. Furthermore, because we can see a clear difference in the stability of oxygen vacancies depending on their surrounding cations, we find that it is not just the concentration of the Ni inclusions but also their distribution that governs vacancy formation and thus photocatalytic activity.

We also considered water adsorption on the La@ZrS_2_ surface, and, in fact, we find that an H_2_O molecule adsorbs readily by coordination to the La adatom through a La–O bond. The adsorption energy of La-bound water is in the order of −1.5 to −1.7 eV in all three systems, exceeding that of neat Co_*x*_Ni_*y*_O­(OH)_*z*_. Another adsorption mode can also be found where the molecule is adsorbed at a Zr top site with the hydrogens parallel to the surface, ostensibly for optimal proximity to the sulfurs. However, in all cases, the dissociation of the H_2_O molecule into H* and OH* on the surface of ZrS_2_ is clearly endothermic by approximately +2.2 eV and thus very unfavorable. Therefore, dissociation on (basal) ZrS_2_ is unlikely without further activating changes to the system.

## Conclusions

We report a computational exploration of La@ZrS_2_/Co_*x*_Ni_*y*_O­(OH)_*z*_ materials and present methods for tuning their properties for the purposes of photocatalysis. Predictable combinations of H, La, and Ni impurities induced a metal-to-semiconductor transition in CoO_2_ monolayer derivatives, where the band edges turned out to be highly tunable with the impurities. With this in mind, semiconducting CoO_2_-based bilayers were designed that included a ZrS_2_ monolayer on Co_*x*_Ni_*y*_O_2_, a van der Waals gap between, and adsorbed H and La atoms at the periphery. DFT simulation of the La@ZrS_2_/Co_*x*_Ni_*y*_O­(OH)_*z*_ systems showed how the electronic states localized in-part on the separate layers and hybridized in-part across the whole bilayer. Overall, ground-state electron density was redistributed from La@ZrS_2_ to Co_*x*_Ni_*y*_O­(OH)_*z*_ to an extent that increased with the La concentration. Accordingly, an interlayer dipole was formed, which contributed to the total dipole across the systems, together with the intralayer dipoles on La@ZrS_2_ and on Co_*x*_Ni_*y*_O­(OH)_*z*_.

The redox potentials of the La@ZrS_2_/Co_*x*_Ni_*y*_O­(OH)_*z*_ bilayers were determined by impurity and interfacial effects. The energetic position of the La@ZrS_2_ conduction band was strongly elevated by bilayer formation, whereas the band edges of Co_*x*_Ni_*y*_O­(OH)_*z*_ hardly changed. This was attributed to separate effects by H and La adsorption and interlayer dipole formation. The VBM on Co_*x*_Ni_*y*_O­(OH)_*z*_ was precisely tuned to alkaline OER at H concentrations of about 5.5 atoms/nm^2^. The CBM localized on La@ZrS_2_ and possessed a suitable energy for alkaline HER at a La concentration of 0.53 atoms/nm^2^. Therefore, LaZr_16_S_32_/Co_14_Ni_14_O_45_(OH)_11_ emerged as the most promising water splitting photocatalyst. In further elaboration, a lower vacancy formation energy on 3-Ni-coordinated oxygen sites suggested that photocatalytic activity is also affected by the local Co–Ni distribution.

This derived end point in specific stoichiometry and nanostructural features may be considered a recommendation for synthetic research. However, it is not meant as a static ultimate goal. The revealed physicochemical insights may be extremely useful in further adjusting and evolving the system proposed here. We believe that this work may spark important developments in the field of photocatalytic water splitting.

## Computational Details

The DFT calculations were performed with VASP software. The Perdew–Burke–Ernzerhof (PBE) exchange-correlation functional was used,^61^ which uses the generalized gradient approximation (GGA). In addition, dispersion interactions were corrected for by the DFT-D3 method with Becke–Johnson damping included.^62^ A Hubbard *U* correction was introduced according to the Dudarev method in the attempt to mitigate DFT’s general issue of excessive delocalization.^63^ The *U*-values on Co, La, Ni, and Zr were chosen at 3.5, 7.5, 5.5, and 5 eV, respectively, based on previous literatures dealing with similar materials.^64–70^ Spin polarization was not included in optimization of the geometries. Still, the electronic structures of the minimum energy geometries were recalculated with collinear spin included. In general, the latter outcomes are presented unless explicitly mentioned otherwise.

GW PBE PAW pseudopotentials from the potpaw.64 library were selected for each element according to the recommendations by the VASP developers.^71,72^ The cutoff energy of the plane wave basis was chosen at 520 eV in all cases. As a rule of thumb, *k*-point density was chosen at $\frac{60}{a}\times \frac{60}{b}\times 1$ , where *a* and *b* are the lattice lengths. Gaussian smearing was used with a smearing width of 0.03 eV to assign occupations to the electronic states. Electronic optimization was carried out over the blocked-Davidson scheme^73,74^ where convergence was assumed when the change in energy over consecutive electronic optimization steps dropped below 10^–8^ eV. Geometries were set to be converged as the change in energy over consecutive geometry optimization steps lowered below 10^–5^ eV.

CoO_2_ and ZrS_2_ unit cells were initially retrieved from the C2DB database (Figure S10).^43,44^ In all constructed slab models, a vacuum space was introduced by setting the lattice length along the *z*-axis at a value of 30 Å. A dipole correction was added along the *z*-axis to compensate for the unwanted dipole field by the periodic images above and below.^75–78^ In the bilayer models, only a single La atom was introduced. The surface concentration of La was varied by changing the size of the model. The three tested variations were made of 4 × 4, 6 × 4, and 8 × 4 ZrS_2_ unit cells on 4 × 7, 6 × 7, and 8 × 7 CoO_2_ unit cells, respectively (Figures 3b and 4b). Thereby, La@ZrS_2_ is orientated at a 19.7 ^0^ twist angle with respect to Co_*x*_Ni_*y*_O­(OH)_*z*_, which is assumed not to critically impact our outcomes. In dimensional analysis of the optimized structures, the lattice parameters of the bilayers were found to deviate between −0.4 and 0.2% compared to the constituting Co_*x*_Ni_*y*_O­(OH)_*z*_ monolayer, and between −0.2 and 1.2% compared to the constituting La@ZrS_2_ monolayer. Hence, local strains on the bilayers were considered negligible. On the other hand, local configurational variability of Co, Ni, and H atoms had to be accepted over bilayers with varied impurity concentrations. In arbitrarily spreading these atomic species in the models, it was generally attempted to pick a most homogeneous distribution.

For the examination of reaction intermediate binding, the chemical potential of water and hydrogen was approximated as their DFT total energy plus a zero-point energy correction calculated using computationally determined vibrational frequencies and the harmonic approximation. The applied ZP corrections were +0.28 eV for H_2_ and +0.57 eV for H_2_O. Adsorption energies for the surface-bound intermediates are then calculated relative to the gas-phase molecules. For determining oxygen vacancy formation energies, the chemical potential of oxygen was estimated as μ_O_ = μ_H_2_O_ – μ_H_2__ and the vacancy formation energy was calculated according to eq 5

$$\Delta E_{\mathrm{Ov}}=E\left[\mathrm{La}@\mathrm{Zr}S_{2}/{\mathrm{Co}}_{x}{\mathrm{Ni}}_{y}O{\left(\mathrm{OH}\right)}_{z}+\mathrm{Ov}\right]+\mu_{O}-E\left[\mathrm{La}@\mathrm{Zr}S_{2}/{\mathrm{Co}}_{x}{\mathrm{Ni}}_{y}O{\left(\mathrm{OH}\right)}_{z}\right]$$ 5

where *E*[La@ZrS_2_/Co_*x*_Ni_*y*_O­(OH)_*z*_] is the total energy of the neat bilayer system and *E*[La@ZrS_2_/Co_*x*_Ni_*y*_O­(OH)_*z*_ + Ov] is the total energy of the bilayer system with the vacancy.

Reaction free energies for water splitting were calculated following the computational hydrogen electrode (CHE) principle, which has been used widely to HER/OER reactions on a variety of materials.^79^ The (electro)­chemical potential of a proton–electron pair at *U* = 0 (relative to the standard hydrogen electrode) is given by

$${\mathrm{\mu ̅}}_{e^{-}}+{\mathrm{\mu ̅}}_{H^{+}}=\frac{1}{2}\mu_{H_{2}}$$ 6

and the CHE reaction free energy is evaluated using eq 7

$$\Delta G_{r}=\Delta G(U=0,\mathrm{pH}=0)-k_{B}T\ln10\cdot \mathrm{pH}-U$$ 7

where Δ*G*(*U* = 0, pH = 0) is the energy difference between reaction products and reagents and *U* is the potential driving the reaction.

## Supplementary Material



## Data Availability

The optimized structures of this study were made openly available in a Zenodo repository at https://doi.org/10.5281/zenodo.18846704.

## Notes

Interplay between spin polarization and the Hubbard *U* correction, tested bilayers, dynamical stability, vacuum energy changes, optical adsorptions, *Z*-plane averaged potential distributions, adsorption geometries, and CoO_2_ and ZrS_2_ unit cell (PDF)
