# Supplementary material for: Balanced Impurity and Interfacial Effects in La-Loaded ZrS2/Co x Ni y O(OH) z Create Key Characteristics for Photocatalytic Water Splitting in DFT Simulation
Source: ACS Phys Chem Au. 2026 Mar 15;6(3):435–45. doi: 10.1021/acsphyschemau.5c00128 (PMC13220200; doi:10.1021/acsphyschemau.5c00128)
Supplement: Supplementary file 1 [file pg5c00128_si_001.pdf]

Supplementary Information: Balanced impurity and interfacial effects in La-loaded $ZrS_2/Co_xNi_yO(OH)_z$ create key characteristics for photocatalytic water splitting in DFT simulation

Joran Celis[a*], *Aku Lempelto*[a], *Wei Cao*[a]

[a]Nano and Molecular Systems Research Unit, Faculty of Science, University of Oulu, FIN-90014, Oulu, Finland

[*]Email: Joran.celis@oulu.fi

Table of Contents

Supplement S1: Interplay between spin-polarization and Hubbard U correction 2
Supplement S2: Table of tested bilayers 5
Supplement S3: Dynamic stability assessment 6
Supplement S4: Vacuum energy changes 8
Supplement S5: Optical adsorptions 9
Supplement S6: Z-plane averaged potential distributions 10
Supplement S7: Adsorption geometries 11
Supplement S8: CoO_2 and ZrS_2 unit cells 13
Bibliography 14

Supplement S1: Interplay between spin-polarization and Hubbard U correction

When including spin-polarization in modelling the $Co_{24}O_{48}$ and derivative systems illustrated in **figure 1**, the sought-after metal-to-semiconductor transition vanished. Then, all systems except for $Co_{24}O_{12}(OH)_{12}$ and $Co_{24}O_{48}La_{10}$ appeared as semiconductors [1,2] (**figure S1**).

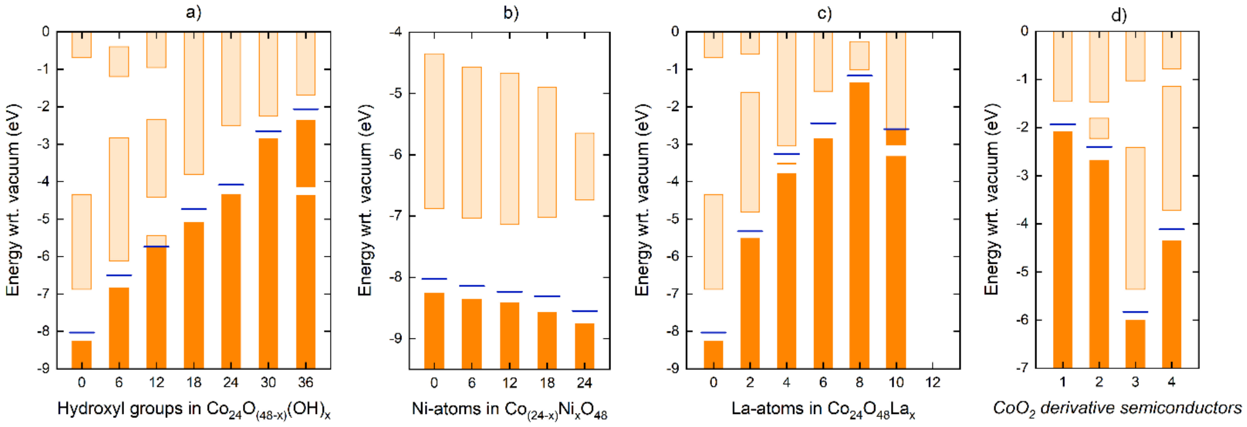

Fig. S1: Schematically represented spin-polarised band structures of CoO_2 monolayer and (a) hydrogen adsorbed, (b) nickel doped, (c) lanthanum adsorbed, and (d) combinatory derivative systems.

We attributed the above tendency towards semiconductivity to an incompatibility between spin-polarization and the Hubbard U correction when modelling metallic CoO_2-derived systems. For the purpose of elaborating, the band structures of $Co_4O_7(OH)_1$, $Co_2Ni_2O_8$ and $Co_4O_4(OH)_4$ were retrieved with increasing U-values on Co and Ni (**figure S3**). Lacking a Hubbard U correction, the valence band on the spin down channel of $Co_4O_7(OH)_1$ and $Co_2Ni_2O_8$ was identified as a single partially occupied cobalt d-band. By gradually introducing an on-site Coulomb interaction, occupied states lowered in energy while unoccupied ones destabilized [3], eventually causing a splitting of the band. As such, the systems appeared as semiconductive. We suspect this feature to be unphysical because it obscured the metal-to-semiconductor transitions given in **figure 1**, which, as mentioned in the article, is supported by experiment [4–8]. Moreover, DFT+U is known to possibly fail describing delocalized metallic systems [9].

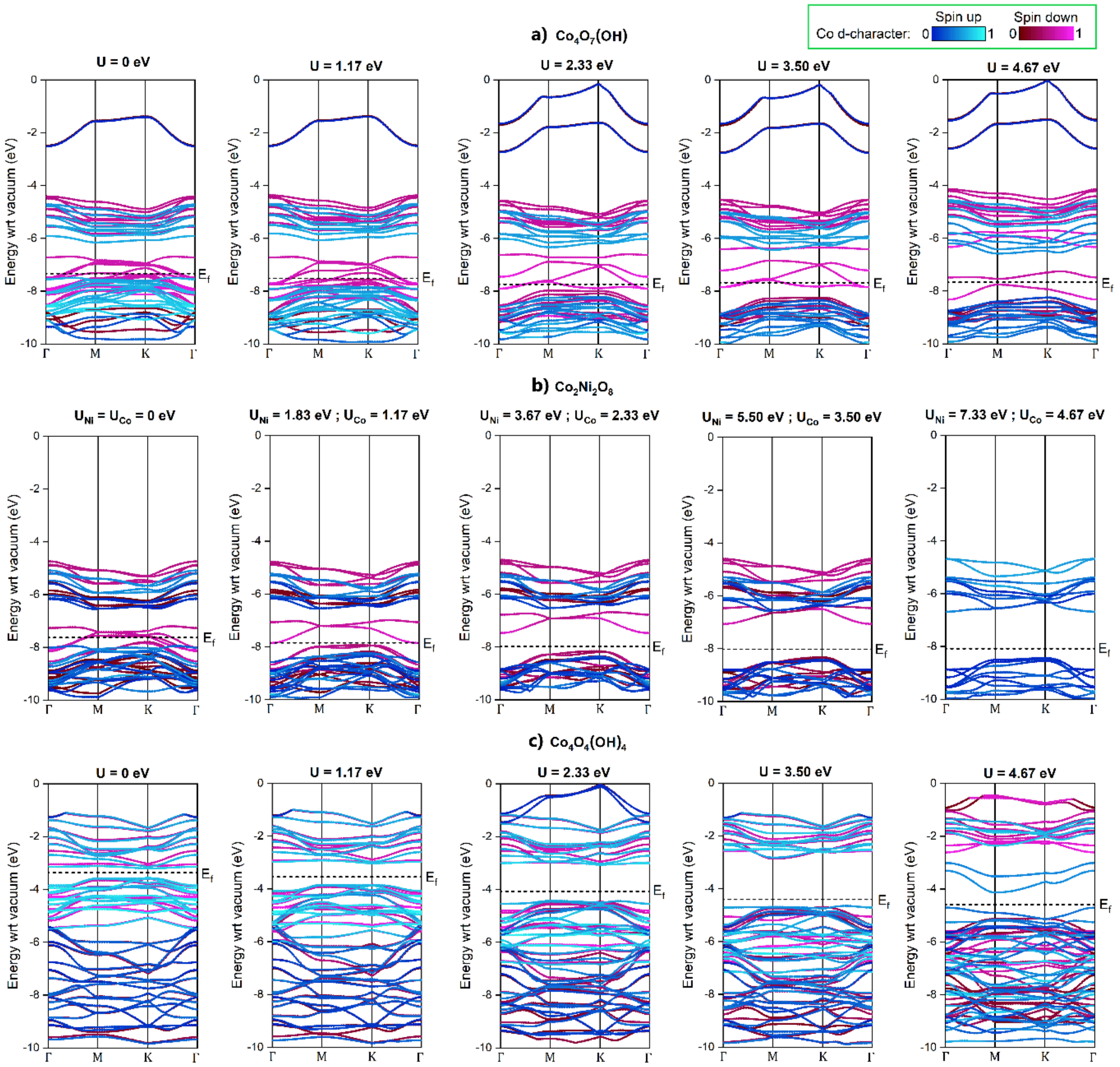

Fig. S2: Band structures of $Co_4O_7(OH)_1$, $Co_2Ni_2O_8$ and $Co_4O_4(OH)_4$ with increasing U-values on Co and Ni atoms. States with higher cobalt d-character were brightened.

When applying a U-correction on semiconductive $Co_4O_4(OH)_4$ (**figure S3c**), (unphysical) d-band splitting did not take place. On the other hand, the U-correction allowed the system's bandgap to be retrieved much more accurately. The bandgap was calculated at 0.21 eV lacking a U-correction, which is a clear underestimation. At a U-value of 3.5 eV, the bandgap opened up to 1.76 eV, which is much closer to the experimental bandgap of 2.4 eV determined by Shi He and coworkers [10]. From this can be concluded that the Hubbard U correction remains sensibly applied in modelling semiconductive CoO_2-derived systems.

For completion, the outcomes given by **figure 1** were recalculated without a U-correction in both non-spin-polarized (**figure S4a-d**) and spin-polarized fashion (**figure S4e-h**). Then, several systems had their bandgap vanish completely in lacking a U-correction, partly obscuring the metal-to-semiconductor transition here as well. Note that here, spin-polarization without U-correction did not create excessive tendency towards semiconductivity. Lastly, a test calculation was carried out on the $Co_{24}O_{24}$ monolayer using the HSE06 functional. The resulting electronic structure conforms to the trend revealed by the calculations without U-corrections and describes the $Co_{24}O_{24}$ as half-metallic unlike spin-polarized GGA+U.

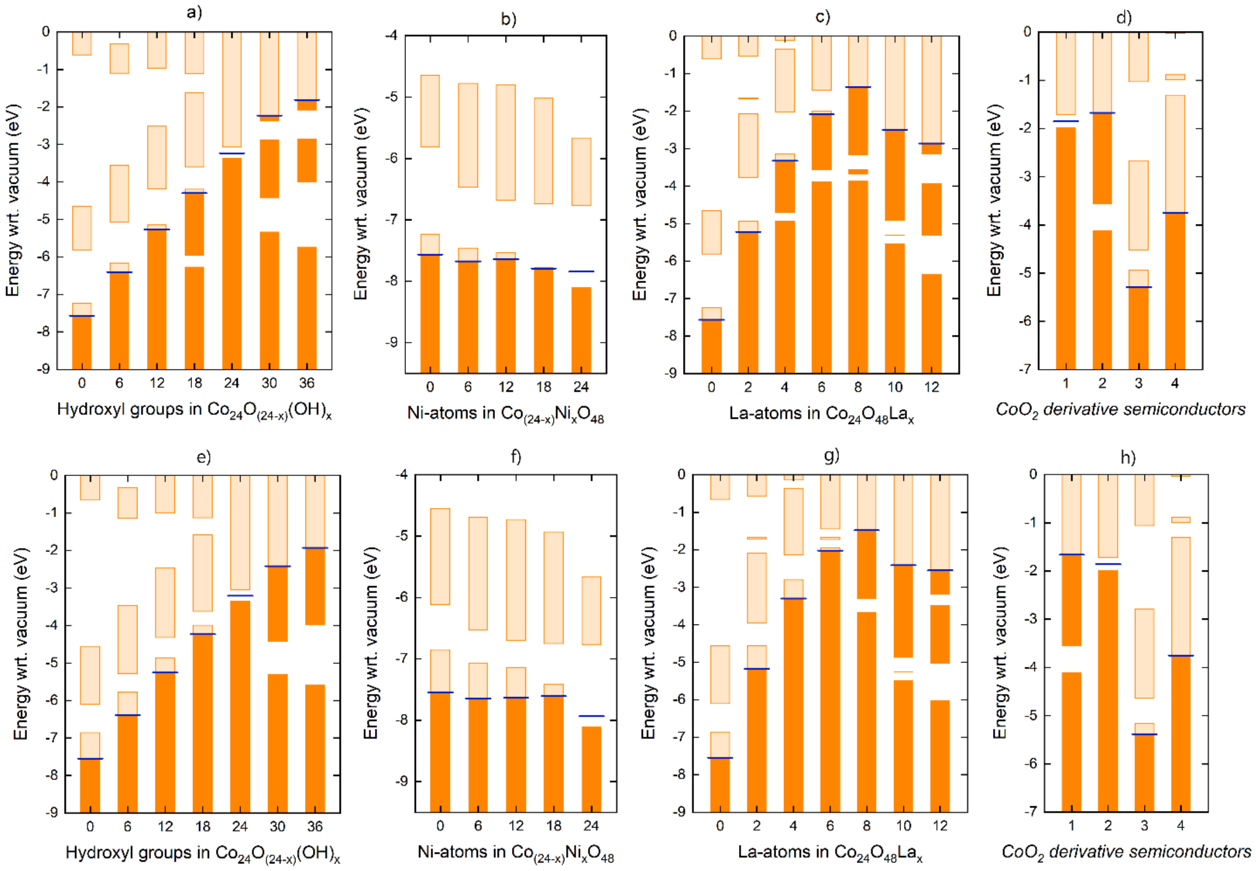

Fig. S3: Schematically represented non-spin-polarized band structures of CoO_2 monolayer and (a) hydrogen adsorbed, (b) nickel doped, (c) lanthanum adsorbed, and (d) combinatory derivative systems and spin-polarized band structures of CoO_2 monolayer and (e) hydrogen adsorbed, (f) nickel doped, (g) lanthanum adsorbed, and (h) combinatory derivative systems. The outcomes were derived without applying a Hubbard U correction.

Supplement S2: Table of tested bilayers

Table S1: stoichiometries and surface concentrations of H and La impurities of the modelled and presented $La@ZrS_2/Co_xNi_yO(OH)_z$ bilayers

Stoichiometry	[H] (atoms/nm^2)	[La] (atoms/nm^2)
$Zr_{16}S_{32}/Ni_{28}O_{56}$	0.00	0.00
$LaZr_{32}S_{64}/Co_{21}Ni_{35}O_{94}(OH)_{18}$	4.72	0.26
$LaZr_{32}S_{64}/Co_{22}Ni_{34}O_{93}(OH)_{19}$	4.98	0.26
$LaZr_{32}S_{64}/Co_{23}Ni_{33}O_{92}(OH)_{20}$	5.27	0.26
$LaZr_{32}S_{64}/Co_{24}Ni_{32}O_{91}(OH)_{21}$	5.52	0.26
$LaZr_{32}S_{64}/Co_{25}Ni_{31}O_{90}(OH)_{22}$	5.78	0.26
$LaZr_{32}S_{64}/Co_{27}Ni_{29}O_{88}(OH)_{24}$	6.30	0.26
$LaZr_{24}S_{48}/Co_{16}Ni_{26}O_{71}(OH)_{13}$	4.54	0.35
$LaZr_{24}S_{48}/Co_{18}Ni_{24}O_{69}(OH)_{15}$	5.24	0.35
$LaZr_{24}S_{48}/Co_{19}Ni_{23}O_{68}(OH)_{16}$	5.58	0.35
$LaZr_{24}S_{48}/Co_{21}Ni_{21}O_{66}(OH)_{18}$	6.27	0.35
$LaZr_{24}S_{48}/Co_{22}Ni_{20}O_{65}(OH)_{19}$	6.62	0.35
$LaZr_{16}S_{32}/Co_{12}Ni_{16}O_{47}(OH)_{9}$	4.74	0.53
$LaZr_{16}S_{32}/Co_{13}Ni_{15}O_{46}(OH)_{10}$	5.26	0.53
$LaZr_{16}S_{32}/Co_{14}Ni_{14}O_{45}(OH)_{11}$	5.78	0.53
$LaZr_{16}S_{32}/Co_{15}Ni_{13}O_{44}(OH)_{12}$	6.29	0.53

Supplement S3: Dynamic stability assessment

Phonon dispersion calculations were performed in an attempt to substantiate the dynamic stability of the investigated bilayers. To this end, the $La@Zr_{16}S_{32}/Co_{14}Ni_{14}O_{45}(OH)_{11}$ bilayer was considered as a representative of the whole set of investigated $La@ZrS_2/Co_xNi_yO(OH)_z$ structures. The finite differences method without symmetry from VASP software package was used, since it is compatible with the simulation settings otherwise used in this research. In consideration of the computational demands, the optimal geometry and the force constants were derived at a single k-point only. The ions were displaced by 0.012 Å in each direction in the computation of the latter. In addition, the macroscopic static dielectric tensor and Born effective charges were calculated based on density functional perturbation theory over a 6×6×1 k-point mesh, to introduce an LO-TO correction to the phonon dispersion, which is only appropriate given that the material is polar.

The phonon dispersions of the $La@Zr_{16}S_{32}/Co_{14}Ni_{14}O_{45}(OH)_{11}$ bilayer and its constituting monolayers are plotted in **figure S4**. Interestingly, the phonon modes of the bilayer do not dip below zero between q-points M and K on the bilayer, in contrast to the monolayers separately. The substantially less profound presence of negative phonon frequencies on the bilayer compared to its constituting monolayers suggests that bilayer formation introduces a stabilizing effect in terms of dynamic stability. Furthermore, the remaining negative phonon frequencies of the bilayer are minor in their magnitude and breadth around Γ. Similar-looking phonon dispersions have been reported for other 2D materials such as α-nitrophosphorene [11], germanene [12], stanene [12] and strained hexagonal boron nitride [13]. In these cases, the negative phonon frequencies were attributed to strain effects or to the implementation of DFT, but not to dynamic instabilities of the material.

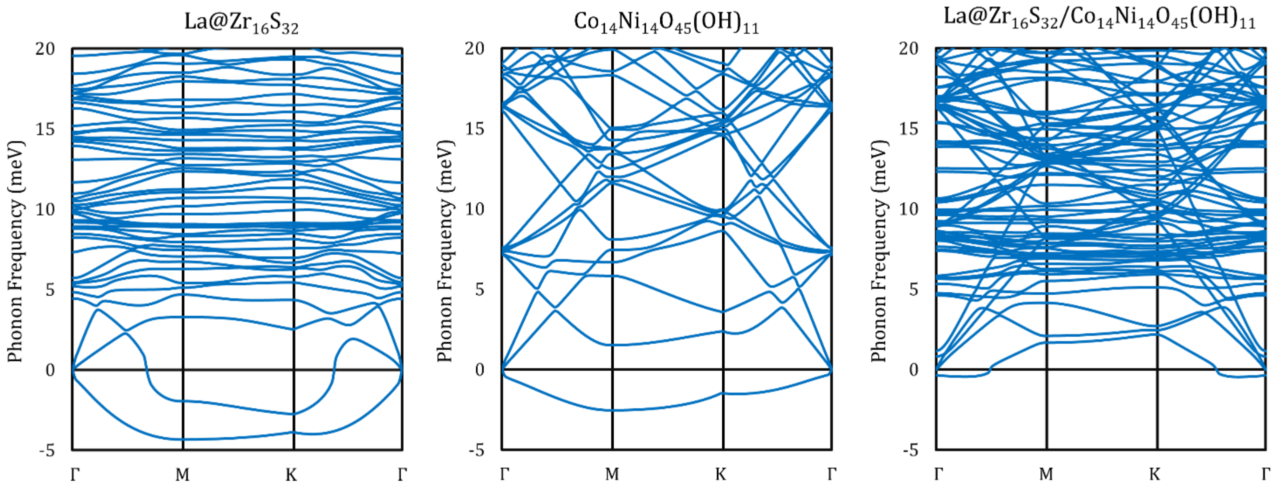

Fig. S4: Phonon dispersions of $La@Zr_{16}S_{32}/Co_{14}Ni_{14}O_{45}(OH)_{11}$ bilayer and its constituting monolayers.

Supplement S4: Vacuum energy changes

A perfect linear relationship between the total dipole moment and the vacuum energy change across the system is apparent from the similarity between **figure 6** and **figure S6**.

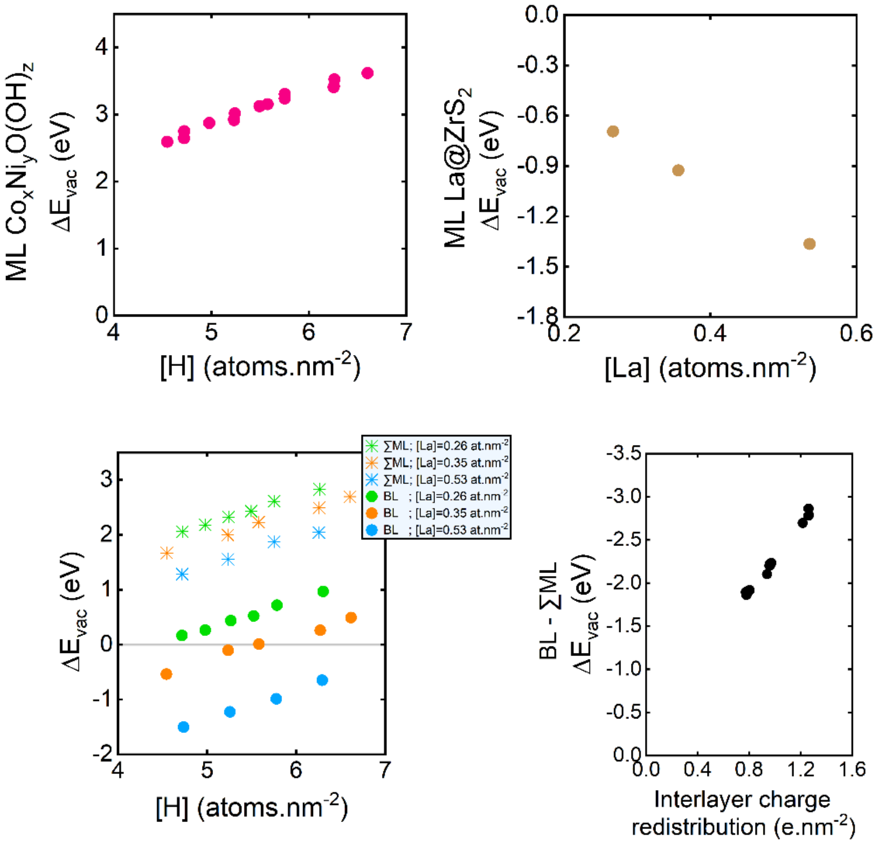

Fig. S5: calculated upward potential change on (a) $Co_xNi_yO(OH)_z$ monolayer (b) La@ZrS_2 monolayer (c) La@ZrS_2/$Co_xNi_yO(OH)_z$ bilayers and (d) calculated upward potential change as a result of interlayer charge transfer in La@ZrS_2/$Co_xNi_yO(OH)_z$ bilayers.

Supplement S5: Optical adsorptions

While the bilayer systems are prohibitively large for time-dependent DFT or GW/BSE methods, we make a rough assessment of the optical characteristics of the material by calculating the absorbance from the frequency-dependent dielectric function $\varepsilon(\omega)$ obtained at the DFT level using Green-Kubo relations as implemented in VASP.

The simulated spectra (**figure S6**) show a broad range of adsorption, starting at low visible frequencies and peaking around 2.7 eV (ca. 450 nm, corresponding to blue light).

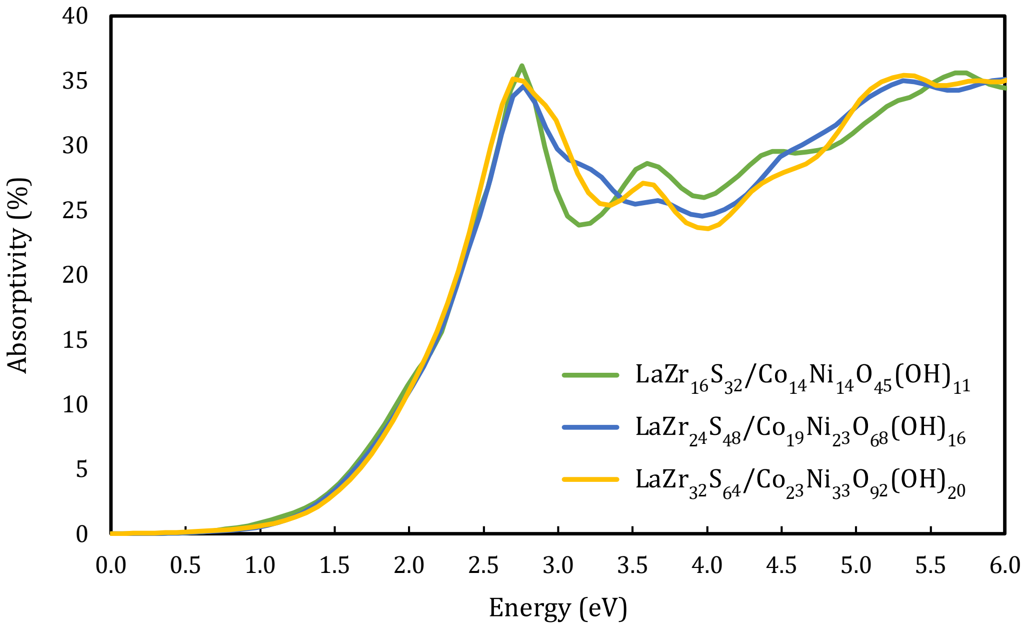

Fig. S6: Simulated photon absorption spectra for $LaZr_{16}S_{32}/Co_{14}Ni_{14}O_{45}(OH)_{11}$, $La@Zr_{24}S_{48}/Co_{19}Ni_{23}O_{68}(OH)_{16}$, and $LaZr_{32}S_{64}/Co_{23}Ni_{33}O_{92}(OH)_{20}$

Supplement S6: Z-plane averaged potential distributions

It was seen how electron density redistribution from La@ZrS_2 to $Co_xNi_yO(OH)_z$ creates an interlayer dipole which is linked to the vacuum energy difference, thus, the electronic potentials at the vacuum regions above and below the system. It was also claimed that the potential lies at the origin of the bending of the conduction band states. Hence, it may be of interest to visually illustrate the z-plane averaged potential over the whole system in an attempt to rigorously confirm the latter. The z-plane averaged potentials of $LaZr_{32}S_{64}/Co_{23}Ni_{33}O_{92}(OH)_{20}$, $LaZr_{24}S_{48}/Co_{19}Ni_{23}O_{68}(OH)_{16}$ and $LaZr_{16}S_{32}/Co_{14}Ni_{14}O_{45}(OH)_{11}$ are given in **Figure S7**. However, no further information becomes immediately apparent from it. Besides the changing vacuum levels, which are already covered in analysis in this work given elsewhere, one could pay attention to the downward peaks in the z-plane averaged potential, but those rather provide information on the positions of the nuclei.

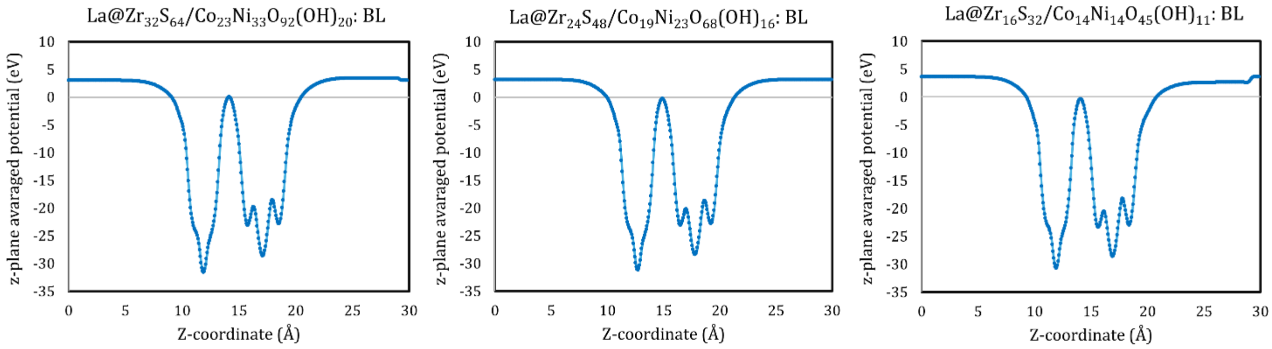

Fig. S7: z-plane averaged potential in three La@ZrS_2/$Co_xNi_yO(OH)_z$ systems.

The component of the z-plane averaged potential which comes forth specifically from interlayer charge redistribution is difficult to properly isolate. Nevertheless, interlayer charge redistribution is a part of the total interlayer interaction. The contribution of the latter to the z-plane averaged potential in the bilayer can be readily obtained. This is calculated by subtracting the z-plane averaged potential of the bilayer with the sum of its separate monolayers. For the three bilayers in question, the outcome is plotted in **figure S8**. Notice that the z-plane averaged potential change due to the interlayer interaction is increasingly negative in the region occupied by La@ZrS_2 as the La concentration

increases and a larger interlayer charge transfer occurs. Implied by the more negative values is that electrons will energetically favor occupation on La@ZrS_2 over $Co_xNi_yO(OH)_z$, which may explain the band bending illustrated in **figure 9**.

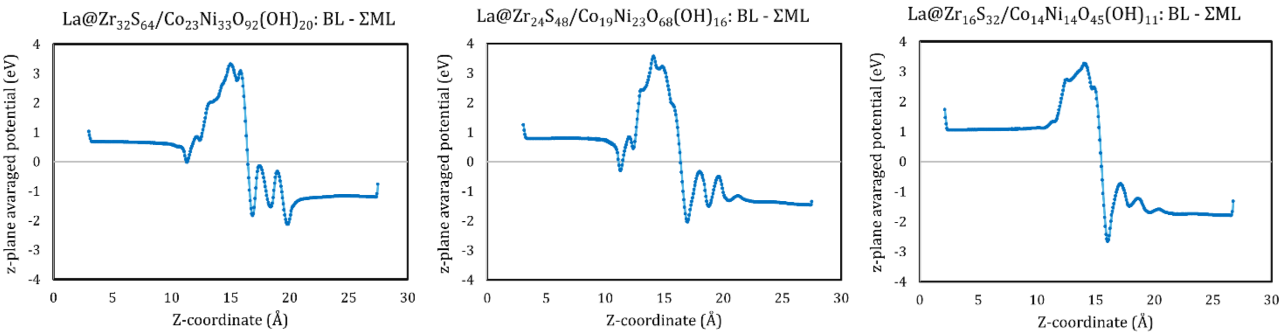

Fig. S8: z-plane averaged potential change due to the interlayer interaction in three La@$ZrS_2/Co_xNi_yO(OH)_z$ systems.

Supplement S7: Adsorption geometries

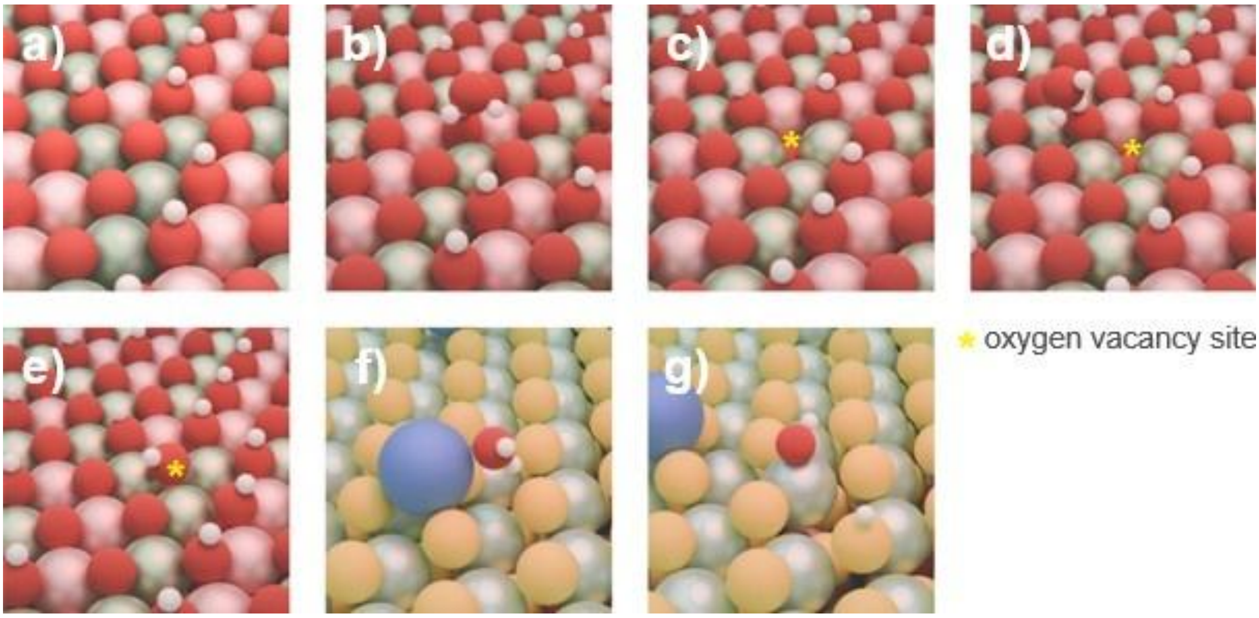

Fig. S9: Adsorption geometries of species on the surface of the La@$Zr_{24}S_{48}/Co_{19}Ni_{23}O_{68}(OH)_{16}$ system: (a) neat $Co_{19}Ni_{23}O_{68}(OH)_{16}$ surface, (b) H_2O on neat $Co_{19}Ni_{23}O_{68}(OH)_{16}$ surface, (c) oxygen vacancy on $Co_{19}Ni_{23}O_{68}(OH)_{16}$ surface, (d) H_2O adsorbed next to oxygen vacancy (e) dissociated H_2O filling the oxygen vacancy, (f) H_2O on La@$Zr_{24}S_{48}$, (g) dissociated H_2O on La@$Zr_{24}S_{48}$.

Table S2: Reaction free energies determined with the CHE approach for water splitting on the surface (hydroxide side) of $LaZr_{24}S_{48}/Co_{19}Ni_{23}O_{68}(OH)_{16}$, calculated using the computational hydrogen electrode approach at T=298 K, U=0.

System	$\Delta G_r(*H_2O \rightarrow *OH + *H^+ + e^-)$, pH=7	$\Delta G_r(*H_2O \rightarrow *OH + *H^+ + e^-)$, pH=14
−4H	2.37	1.96
−6H	1.95	1.54
−8H	2.11	1.70
−10H	2.34	1.93
−12H	1.10	0.68
−14H	1.61	1.20
−16H	1.33	0.91
Oxygen vacancy	0.60	0.18

Table S3: DFT-calculated energies for consecutive hydrogen removal (H_2 de-adsorption) from $LaZr_{24}S_{48}/Co_{19}Ni_{23}O_{68}(OH)_{16}$, as well as the adsorption energies (on the hydroxide side) of molecular and dissociated water (at T=0 K).

System	$\Delta E_{H_2\ deads.}$ (eV)	$\Delta E_{ads}(H_2O)$ (eV)	$\Delta E_{ads}(OH + H)$ (eV)	$\Delta E(*H_2O \rightarrow *OH + *H)$ (eV)	$\Delta E_{ads}(*OH)^{\dagger}$ (eV)
Neat surface	—	−1.28	recombines	—	recombines
−2H	2.78	−1.12	recombines	—	recombines
−4H	3.04	−1.11	−0.15	0.96	1.68
−6H	3.04	−0.87	−0.17	0.71	1.49
−8H	3.19	−0.72	−0.02	0.70	1.81
−10H	3.48	−1.16	−0.25	0.91	1.60
−12H	3.59	−0.51	−0.83	−0.32	1.00
−14H	3.39	−1.18	−1.16	0.03	0.85
−16H	2.35	−0.32	0.15	0.47	1.42
Oxygen vacancy	—	−0.95	−0.93	0.02	0.06

† $E_{ads}(*OH)$ calculated as a $\left[E(*OH) + E\left(\frac{1}{2}H_2\right)\right] - [E(*) + E(H_2O)]$

Supplement S8: CoO_2 and ZrS_2 unit cells

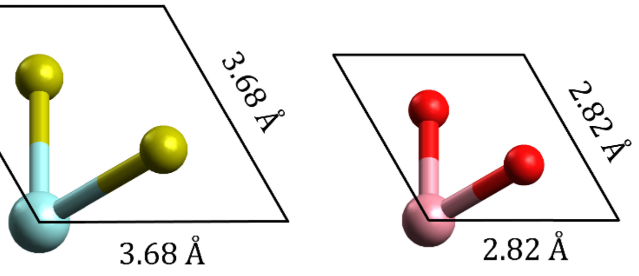

Fig. S10: top views of ZrS_2 unit cell (left) and CoO_2 unit cell (right)

Bibliography

(1) Xu, X. L.; Hu, C. E.; Wu, H. J.; Geng, H. Y.; Chen, X. R. Magnetothermal Properties of CoO2 Monolayer from First-Principles and Monte Carlo Simulations. *J. Appl. Phys.* **2024**, *135* (21). https://doi.org/10.1063/5.0207379.

(2) Liang, L.; Du, S.; Wang, L.; Liu, Z.; Wu, J.; Zhang, S. Tunable Magnetic and Electronic Properties of the 2D CoO2Layer. *Journal of Physical Chemistry C* **2021**, *125* (1), 873–877. https://doi.org/10.1021/acs.jpcc.0c07847.

(3) Kulik, H. J. Perspective: Treating Electron over-Delocalization with the DFT+U Method. *Journal of Chemical Physics* **2015**, *142* (24). https://doi.org/10.1063/1.4922693.

(4) Foo, M. L.; Wang, Y.; Watauchi, S.; Zandbergen, H. W.; He, T.; Cava, R. J.; Ong, N. P. Charge Ordering, Commensurability, and Metallicity in the Phase Diagram of the Layered NaxCoO2. *Phys. Rev. Lett.* **2004**, *92* (24). https://doi.org/10.1103/PhysRevLett.92.247001.

(5) Nguyen, D. L.; Hsing, C. R.; Wei, C. M. Theoretical Prediction of Superconductivity in Monolayer CoO2. *Nanoscale* **2019**, *11* (36), 17052–17057. https://doi.org/10.1039/c9nr03954f.

(6) Nadkarni, N.; Zhou, T.; Fraggedakis, D.; Gao, T.; Bazant, M. Z. Modeling the Metal–Insulator Phase Transition in LixCoO2 for Energy and Information Storage. *Adv. Funct. Mater.* **2019**, *29* (40). https://doi.org/10.1002/adfm.201902821.

(7) Milewska, A.; Świerczek, K.; Tobola, J.; Boudoire, F.; Hu, Y.; Bora, D. K.; Mun, B. S.; Braun, A.; Molenda, J. The Nature of the Nonmetal-Metal Transition in LixCoO 2 Oxide. In *Solid State Ionics*; Elsevier, 2014; Vol. 263, pp 110–118. https://doi.org/10.1016/j.ssi.2014.05.011.

(8) Marianetti, C. A.; Kotliar, G.; Ceder, G. A First-Order Mott Transition in LixCoO2. *Nat. Mater.* **2004**, *3* (9), 627–631. https://doi.org/10.1038/nmat1178.

(9) Tolba, S. A.; Gameel, K. M.; Ali, B. A.; Almossalami, H. A.; Allam, N. K. The DFT+U: Approaches, Accuracy, and Applications. In *Density Functional Calculations - Recent Progresses of Theory and Application*; InTech, 2018. https://doi.org/10.5772/intechopen.72020.

(10) He, S.; Huang, Y.; Huang, J.; Liu, W.; Yao, T.; Jiang, S.; Tang, F.; Liu, J.; Hu, F.; Pan, Z.; Liu, Q. Ultrathin CoOOH Oxides Nanosheets Realizing Efficient Photocatalytic Hydrogen Evolution. *Journal of Physical Chemistry C* **2015**, *119* (47), 26362–26366. https://doi.org/10.1021/acs.jpcc.5b09442.

(11) Taheri, A.; Pisana, S.; Singh, C. V. Importance of Quadratic Dispersion in Acoustic Flexural Phonons for Thermal Transport of Two-Dimensional Materials. *Phys. Rev. B* **2021**, *103* (23). https://doi.org/10.1103/physrevb.103.235426.

(12) Kuang, Y. D.; Lindsay, L.; Shi, S. Q.; Zheng, G. P. Tensile Strains Give Rise to Strong Size Effects for Thermal Conductivities of Silicene, Germanene and Stanene. *Nanoscale* **2016**, *8* (6), 3760–3767. https://doi.org/10.1039/c5nr08231e.

(13) Anees, P.; Valsakumar, M. C.; Panigrahi, B. K. Effect of Strong Phonon-Phonon Coupling on the Temperature Dependent Structural Stability and Frequency Shift of 2D Hexagonal Boron Nitride. *Physical Chemistry Chemical Physics* **2016**, *18* (4), 2672–2681. https://doi.org/10.1039/c5cp06111c.
